# Mapping roadless areas in regions with contrasting human footprint

**DOI:** 10.1038/s41598-024-55283-3

**Published:** 2024-02-27

**Authors:** Monika T. Hoffmann, Katarzyna Ostapowicz, Kamil Bartoń, Pierre L. Ibisch, Nuria Selva

**Affiliations:** 1 Institute of Nature Conservation, Polish Academy of Sciences, 31-120 Krakow, Poland; 2 Norwegian Institute of Nature Research (NINA), FRAM-High North Centre for Climate and the Environment, 9296 Tromsø, Norway; 3 Institute of Geography and Spatial Management, Faculty of Geography and Geology, Jagiellonian University, 30-387 Krakow, Poland; 4 Centre for Econics and Ecosystem Management, Eberswalde University for Sustainable Development, 16225 Eberswalde, Germany; 5 Departamento de Ciencias Integradas, Facultad de Ciencias Experimentales, Centro de Estudios Avanzados en Física, Matemáticas y Computación, Universidad de Huelva, 21071 Huelva, Spain; 6 Estación Biológica de Doñana, Consejo Superior de Investigaciones Científicas, 41092 Sevilla, Spain

**Keywords:** Roadless areas, OpenStreetMap, Road mapping, Road ecology, Anthropogenic impact, Human footprint index, Human modification index, Travel time to major cities, Conservation biology, Environmental impact

## Abstract

In an increasingly human- and road-dominated world, the preservation of functional ecosystems has become highly relevant. While the negative ecological impacts of roads on ecosystems are numerous and well documented, roadless areas have been proposed as proxy for functional ecosystems. However, their potential remains underexplored, partly due to the incomplete mapping of roads. We assessed the accuracy of roadless areas identification using freely available road-data in two regions with contrasting levels of anthropogenic influence: boreal Canada and temperate Central Europe (Poland, Slovakia, Czechia, and Hungary). Within randomly selected circular plots (per region and country), we visually examined the completeness of road mapping using OpenStreetMap 2020 and assessed whether human influences affect mapping quality using four variables. In boreal Canada, roads were completely mapped in 3% of the plots, compared to 40% in Central Europe. Lower Human Footprint Index and road density values were related to greater incompleteness in road mapping. Roadless areas, defined as areas at least 1 km away from any road, covered 85% of the surface in boreal Canada (mean size ± s.d. = 272 ± 12,197 km^2^), compared to only 0.4% in temperate Central Europe (mean size ± s.d. = 0.6 ± 3.1 km^2^). By visually interpreting and manually adding unmapped roads in 30 randomly selected roadless areas from each study country, we observed a similar reduction in roadless surface in both Canada and Central Europe (27% vs 28%) when all roads were included. This study highlights the urgent need for improved road mapping techniques to support research on roadless areas as conservation targets and surrogates of functional ecosystems.

## Introduction

Habitat fragmentation, one of the greatest threats to biodiversity^1^, has already altered more than 50% of the Earth's terrestrial landscapes^2^, with the road network emerging as a major driver of ecosystem fragmentation and degradation^3^. Its effects on the environment are numerous, including defaunation, deforestation, land use changes, and urban sprawl. These factors collectively drive the loss of biodiversity and ecosystem functionality i. e. the capacity of ecosystems to sustain essential ecological processes and services over time^3–6^. Other ecological impacts of roads include pollution, soil erosion, isolation of populations, alterations in species behavior, wildlife mortality, changes in gene flow, facilitation of invasive species and increase in fire risk^5,7–10^. The intensity of these impacts varies based on factors such as road surface, density, location, type, and traffic volume^11,12^. While road development is often associated with economic growth and urbanization^13,14^, its environmental impacts may not always align with sustainable development and its goals^3,15,16^. Road construction continues to meet the growing demand of natural resources by providing access to unexploited regions and facilitating resource extraction^4^. Especially in pristine and natural areas, the consequences of road construction and the following contagious development may have a catastrophic effect on ecosystems^5,17^. In recent years, road networks have penetrated areas previously considered remote and devoid of human infrastructure, leading to unprecedented accessibility^11,18^. Growing evidence emphasizes that roads disrupt and degrade the functionality of ecosystems^3^. Therefore, it is crucial to identify the remaining areas still unfragmented by roads and prevent the first cut into these functioning ecosystems and the subsequent contagious development^5^.

In the current global biodiversity and climate crisis, understanding the extent and condition of unfragmented regions and their role in biodiversity conservation is critical to maintaining ecosystem resilience at different scales^19^. Roadless areas are relatively free from all human impacts associated with the road network and have been proposed as conservation targets for functional ecosystems^3,5,9,20^. To protect them effectively, they must first be accurately identified. This is not only essential but also urgent, given the current pace of road construction^21,22^. Roadless areas can serve as quantifiable indicators for the most pristine ecosystems and can play an important role in maintaining ecosystem functions and contributing to biodiversity and ecological processes^9,23^. They facilitate species movement, long-distance dispersal, and increase connectivity among ecoregions^24^. Roadless areas have a greater buffering capacity and are more resilient than fragmented areas to the impacts of climate change^3,25^.

It has been estimated that the length of paved roads will increase by 14–23%^26^ or even to 59%^21^ by 2050, therefore, many current roadless areas are likely to disappear before they have even been mapped. Accurate and up-to-date road mapping is urgent but presents significant challenges due to the continuous expansion of roads, the multitude of road types with varying surface reflectance, the extensive length of road networks, the limited accessibility to some road data, and the proliferation of illegal and undocumented roads, particularly notable in regions such as the Amazon basin^11^. The availability and accessibility of high-resolution satellite imagery can support accurate road mapping, but it is also a critical component as it varies around the world. In some regions, imagery can be limited or outdated, affecting the accuracy of road mapping^27^. Environmental conditions such as dense forests, deserts or mountainous terrain can hinder the visibility of roads, making mapping in these areas more challenging. Diverse road types, such as paved, unpaved, forest, or desert roads, have unique characteristics and require different mapping approaches^28,29^. The complexity increases when attempting to differentiate and accurately represent various road surfaces and terrains^30^. This requires sophisticated data processing methods to handle the large amount of data and numerous vertex points involved. On a global level, open-source road datasets are provided by the Center for International Earth Science Information Network (CIESIN), the Global Roads Inventory Project (GRIP) dataset, and the volunteer-based geographic information OpenStreetMap (OSM) road dataset^26,31,32^. For now, OSM is the most complete, up-to-date, and freely available road dataset on a global scale and is constantly being improved^3,33^. However, in some regions, OSM road data does not reflect the full extent of existing roads^3,34,35^, especially in regions of conservation value holding valuable natural resources, where the construction of new roads is a constant threat^16^.

Our goal was to evaluate to what extent roadless areas can be accurately identified and characterized with OSM road data and to assess the completeness of road mapping in two study regions with a priori contrasting road densities—boreal Canada (covering approximately 5.4 million km^2^) and a region in temperate Central Europe covering Poland, Slovakia, Czechia, and Hungary (approximately 533,000 km^2^). The selection of these study regions was based on their contrasting human footprint. Boreal Canada represents a vast wilderness with relatively low human population density and infrastructure development, in contrast to the densely populated and heavily modified landscapes of temperate Central Europe. We expected considerable differences in the completeness of road mapping as well as in the number and size of roadless areas between the two regions related to varying levels of anthropogenic influence. We predicted more incomplete road mapping with lower human footprint, i.e. in areas where significant roadless areas may still remain. We aimed to (a) identify the number and surface of roadless areas in these regions using OSM road data, (b) assess the completeness of OSM road data and to examine the potential effect of anthropogenic influences on road mapping completeness, and (c) compare the quantity and size of roadless areas in the two study regions using OSM road data and road mapping through visual interpretation of high-resolution satellite imagery.

## Results

### Mapping accuracy and associated factors

**Figure 1:**
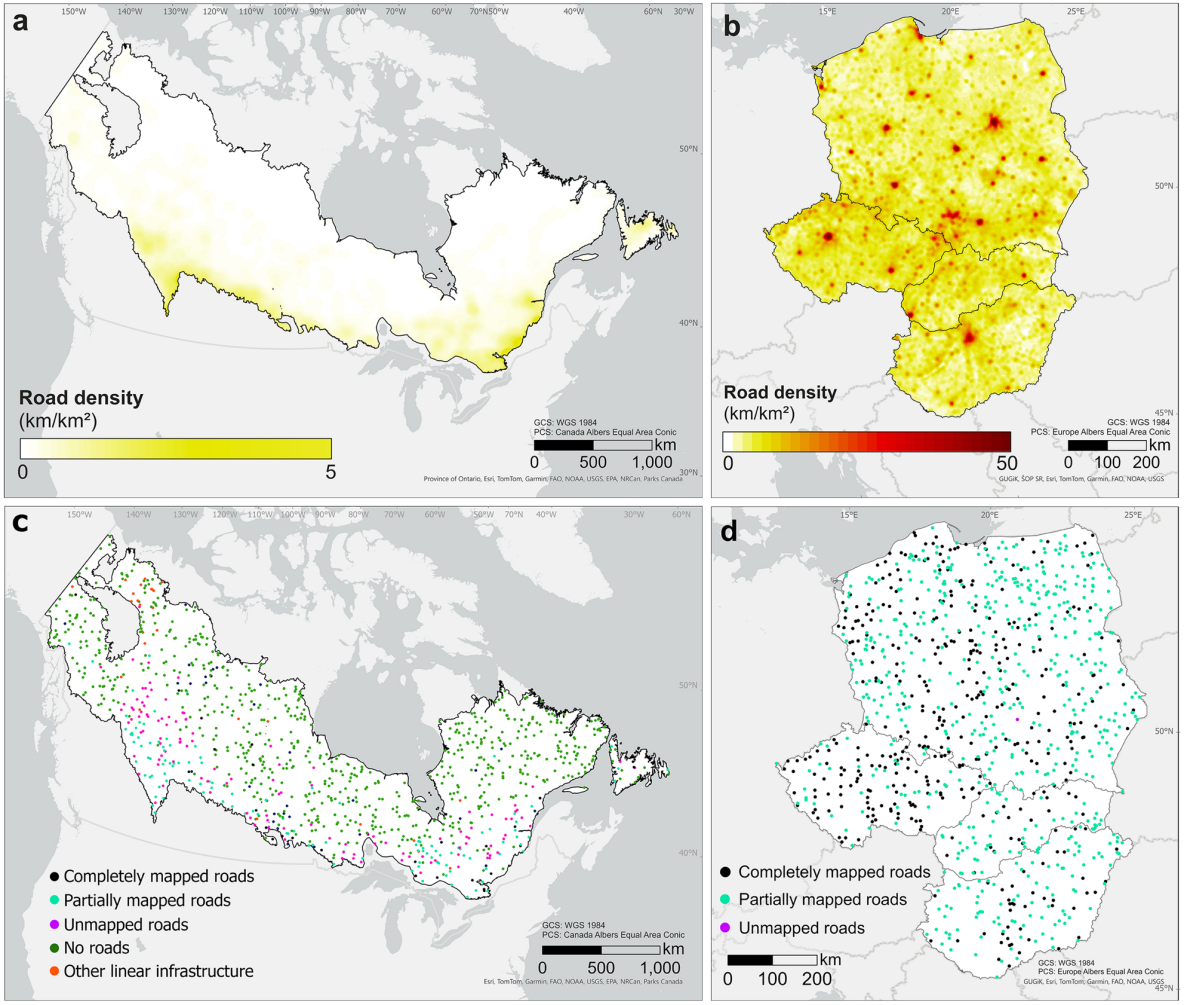
Road density and spatial distribution of the 1000 randomly selected circular plots in each of the two study areas (**a,c**) the boreal region of Canada and (**b,d**) a selected region in temperate Central Europe covering Poland, Slovakia, Czechia, and Hungary. Road densities were estimated using all OSM 2020 road categories and a 5 km^2^ snap raster (**a,b**). Circular plots of 1 km radius were randomly selected and classified after the visual interpretation into the following categories: plots with completely mapped roads, with partially mapped roads, with only unmapped roads, without roads, and with other linear infrastructures (**c,d**). This figure was created using ArcGIS Pro 3.2 (https://www.esri.com/en-us/arcgis/products/arcgis-pro/overview).

According to OSM, the road density in the study region of temperate Central Europe was 11 times higher than in boreal Canada, with a maximum of 41.5 km/km^2^ and 3.9 km/km^2^, respectively. The mean road density was 3.5 km/km^2^ (± 2.4 s.d.) in Central Europe and 0.1 km/km^2^ (± 0.2 s.d.) for boreal Canada (Fig. 1a, b). There was a spatial pattern of increasing road density in boreal Canada from north to south (Fig. 1a). In the study region of temperate Central Europe, the main cities were clearly recognizable due to their high road density, but no latitudinal patterns were observed (Fig. 1b). Road length in both study areas highly varied between the OSM and the Global Roads Inventory Project road datasets; the latter contained only 23% of the OSM road length in the region of temperate Central Europe and 12% in the boreal region of Canada (Table S1). In general, OSM had the longest road network, also when compared with regional road datasets.

The visual interpretation of the randomly selected circular plots (n = 1000 per region, 3.14 km^2^ each) confirmed that 70% of the circular plots in boreal Canada had no roads, while only 3% of the plots had all roads completely mapped by OSM users, making a total of 73% of the plots properly mapped. However, in 12% of the plots roads were partially mapped, i.e., not all road sections were included in OSM, and in another 12%, the plots contained only unmapped roads (Table 1, Fig. 1c). This results in 24% of the plots missing roads in the OSM road dataset for boreal Canada. Out of the 271 plots containing roads, only 11% were properly mapped. In the Central European region, the visual interpretation confirmed that there were no plots without roads and that 40% of the plots were accurately mapped, while 60% of the plots contained partially unmapped roads within the OSM road dataset. Notably, only one plot contained unmapped roads (Table 1, Fig. 1d). To explore potential variations in road mapping across different European countries, we created an additional set of circular plots (n = 4000, 1000 plots per country), and our findings demonstrated that Czechia exhibited the highest percentage of plots with correctly mapped OSM roads, whereas Slovakia had the lowest percentage. Most plots in Central Europe were partially mapped. However, examining individual countries, completely mapped roads emerged as the most frequent category (Table 1, Table S2).

**Table 1:** Summary of the visual interpretation of the randomly selected circular plots (n = 1000, 3.14 km^2^ each) in each of the two study regions (boreal Canada and temperate Central Europe including Poland, Slovakia, Czechia, and Hungary).

Plot categories	Completely mapped roads	Partially mapped roads	Unmapped roads	No roads	Other linear infrastructures
Boreal Canada					
No. plots	31	119	121	703	26
Road density (km/km^2^)	0.4 ± 0.3	0.4 ± 0.3	0.2 ± 0.2	0.0 ± 0.1	0.1 ± 0.1
Travel time to major cities (min)	544.4 ± 338.6	428.8 ± 325.6	763.4 ± 549.4	1737.0 ± 873.2	2067 ± 1164.7
Human footprint index	2.0 ± 3.3	3.6 ± 6.2	0.5 ± 1.6	0.1 ± 0.7	0.2 ± 0.8
Human modification index	0.0 ± 0.1	0.1 ± 0.2	0.0 ± 0.1	0.00 ± 0.0	0.0 ± 0.0
Central Europe					
No. plots	399	600	1	0	0
Road density (km/km^2^)	4.1 ± 2.9	3.2 ± 1.8	–	–	–
Travel time to major cities (min)	77.9 ± 62.5	86.9 ± 60.7	–	–	–
Human footprint index	18.1 ± 9.8	15 ± 8.2	–	–	–
Human modification index	0.6 ± 0.2	0.5 ± 0.2	–	–	–

It shows the number of circular plots within the following categories: plots with all roads completely mapped, plots with roads partially mapped, plots with all roads unmapped, plots without roads, and plots containing other linear infrastructures. The table shows the mean ± s.d. values of road density (km/km^2^), travel time to the nearest city of 50,000 or more people (minutes), Human Footprint Index (ranging from 0 to 50, low values indicated low human footprint), and Human Modification Index (ranging from 0 to 1, low values indicated low degree of landscape modification by humans).

Road-free plots were mainly located in the northern part of boreal Canada (Fig. 1c). Some linear infrastructures were detected in the north-western part of the Canadian study region, including powerlines, seismic lines for oil and gas exploration, and fire breaks, intended to control wildfires. Most of the plots with unmapped and partially unmapped roads were detected in the southern and central parts of boreal Canada. In temperate Central Europe, we did not identify any plot without roads. Plots with partially mapped roads were the most common, followed by plots with completely mapped roads; only one plot had only unmapped roads (Fig. 1d, Table 1).

We investigated the relationship between the completeness of road mapping and different proxies of human impact in the selected plots at the country level (boreal Canada, Poland, Slovakia, Czechia, Hungary): road density, travel time to major cities, the Human Footprint Index, and the Human Modification Index^32,36–38^.

In both regions, there was a negative correlation between road completeness and road density, although the correlation was lower in Central Europe, indicating that higher road density was associated with more comprehensive road mapping. There was also a strong negative correlation between road density and travel time to major cities in Boreal Canada, which was lower in Central Europe. The Human Footprint Index and the Human Modification Index both showed a positive association with road density, while the two indices were moderately correlated (Table 1, Table S3, Fig. S1).

**Figure 2:**
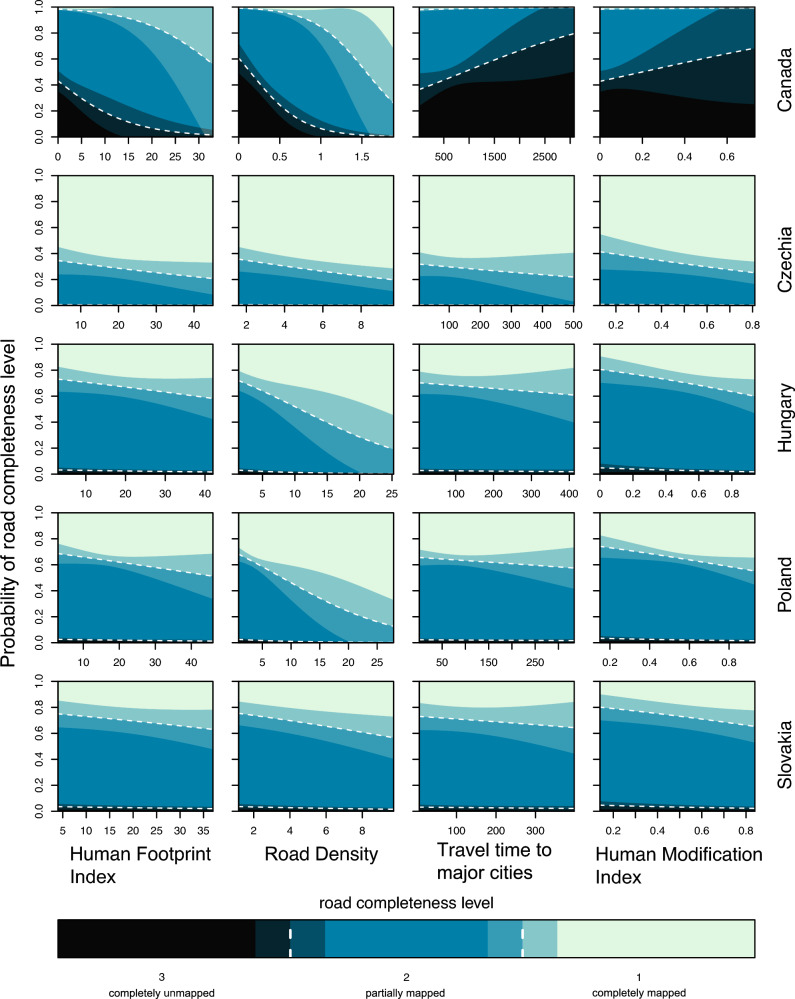
Proportion of plots with three categories of road completeness (completely mapped = 1, partially mapped = 2, completely unmapped = 3), as predicted by the ordinal regression model, in relation to the four variables of anthropogenic influence in each of the five study countries. Shaded regions represent the levels of completeness. Mean values are shown by dashed lines, and intermediate shading indicates 95% confidence intervals.

Accounting for spatial autocorrelation in the data had little effect on the coefficient values, as shown by the comparison of alternative Generalized Least Squares models (Fig. S2). Including the spatial correlation structure only moderately improved the model fit (Δ AIC_*c*_ = 3.3, Tables S4). The Ordinal Regression model showed that road completeness was differently associated with the explanatory variables in the two regions (Table 2). Based on the AIC_*c*_, including the differences between the European countries did not improve the model (Table S5). Human Footprint Index and road density were the most significant predictors overall, and the effect of travel time to major cities and Human Modification Index varied between region (Table 2). The effects of the Human Footprint Index and road density were positive throughout, i.e., the proportion of correctly mapped plots (only plots containing roads were considered) increased as these variables increased, but the effect of the former was lower in the European region. In contrast, travel time to major cities and Human Modification Index had a negative effect in mapping completeness in boreal Canada and a weak positive effect in the Central European countries (Fig. 2).

**Table 2:** Analysis of deviance table (type II tests) for the ordinal regression model of road completeness (completely mapped = 1, partially mapped = 2, completely unmapped = 3).

Model term	x^2^	Df	p value
Region	222.1	1	< 0.0001
Human Footprint Index	19.0	1	< 0.0001
Road density	14.0	1	0.0002
Travel time to major cities	4.6	1	0.03
Human modification index	8.1	1	0.004
Country	51.1	4	< 0.0001
Country × Human footprint index	2.7	1	0.1
Country × Travel time to major cities	5.4	1	0.02
Country × Human modification index	18.9	1	< 0.0001

The columns show model terms, x^2^ test value with degree of freedom, and associated p-value. The reference region and country was boreal Canada.

### Identification, characterization, and mapping of roadless areas

**Figure 3:**
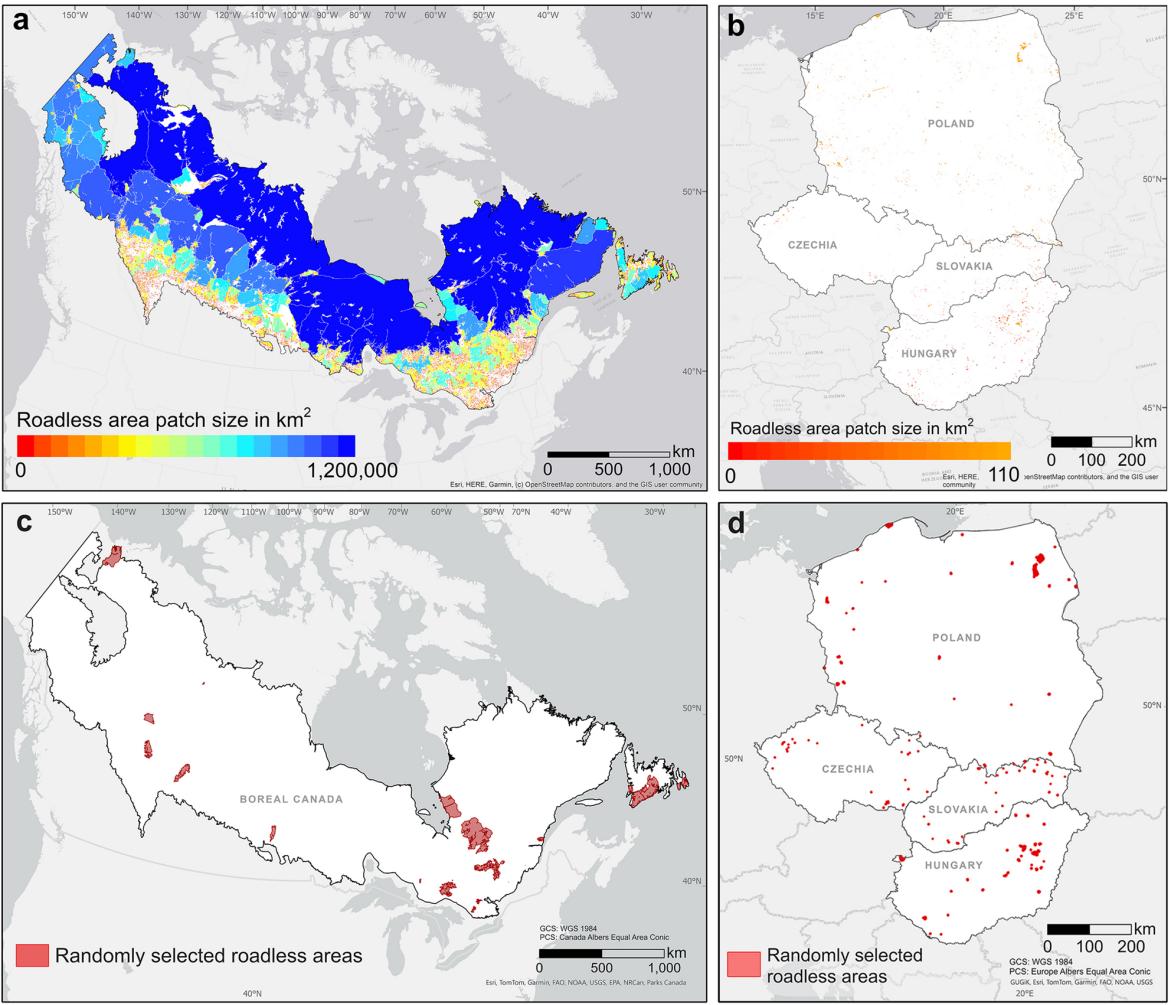
Distribution of roadless areas and their sizes (km^2^). Roadless areas were identified based on a 1 km buffer on each side of every road, for (**a**) the boreal region of Canada, and (**b**) a selected region of temperate Central Europe represented by Poland, Slovakia, Czechia, and Hungary. The spatial distribution of the 30 randomly selected roadless areas (**c**) in the boreal region of Canada, and (**d**) in each of the four selected countries of temperate Central Europe. For more detailed views, in the Supplementary Material Fig. S4 and Fig. S5 provide enlarged versions of (**c,d**), indicating the randomly selected roadless areas with the corresponding numbers. This figure was created using ArcGIS Pro 3.2 (https://www.esri.com/en-us/arcgis/products/arcgis-pro/overview).

In total, 16,786 roadless areas were identified in the boreal region of Canada using the OSM 2020 road dataset. Overall, 85% of the surface of boreal Canada was roadless, with an average patch size of 272 km^2^ but a median size of 0.7 km^2^ (Fig. 3a, Table 3). Over half of the identified roadless areas (54%, 9,112 patches) were smaller than 1 km^2^, and less than 5% (821 roadless patches) were larger than 100 km^2^ (Fig. 3a, Table 3, Table S6).

**Table 3:** Extent and amount of roadless areas for the two study regions boreal Canada and temperate Central Europe using the OSM road dataset (2020).

Identified roadless areas	Boreal Canada	Temperate Central Europe
Study region surface (km^2^)	5,432,563	532,983
No. roadless areas identified	16,786	3,524
Roadless surface (%)	84.5	0.4
Roadless surface (km^2^)	4,560,608.3	2,160.6
Mean size of roadless areas (km^2^)	271.7	0.6
Median size of roadless areas (km^2^)	0.72	0.1
Maximum size of roadless areas (km^2^)	1,173,889	106.8
No. roadless areas (0, 1] km^2^	9,112	3,062
No. roadless areas (1, 10] km^2^	4,762	442
No. roadless areas (10, 50] km^2^	1,673	19
No. roadless areas (50, 100] km^2^	418	3
No. roadless areas (100, 1,173,889] km^2^	821	1
Selected roadless areas for visual interpretation and road mapping		
No. roadless areas (before)	30	120
No. roadless areas (after)	1408	100
Mean size of roadless areas (before) (km^2^)	4223	4.9
Mean size of roadless areas (after) (km^2^)	58	4.2
Total roadless surface (before) (km^2^)	127,199.5	586.6
Total roadless surface (after) (km^2^)	93,497.6	422.8
Roadless surface lost (%)	33,701.9 (26.5)	164.1 (27.97)
No. original roadless areas lost	1	20
Length of roads manually mapped (km)	34,787	257

Roadless areas were calculated by creating a 1 km geodesic buffer around each road and extracting the remaining area. The table provides information on the number of roadless areas in different size classes, along with the mean, median, and maximum size, as well as the total roadless surface as the sum of all roadless areas patches and the corresponding percentage of the region surface**.** The second part of the table includes similar metrics for the 30 randomly selected areas before and after visual interpretation and manual road mapping in both regions.

Our data highlight the substantial difference in the extent and size of roadless areas between the two regions, with boreal Canada having a much larger percentage of roadless areas, including some substantial patches, compared to Central Europe, where roadless areas were relatively small and accounted for only a small proportion of the total area (Table 3, Fig. 3a, b). The total number of roadless areas in Central Europe was 3,524. Only 0.4% (2161 km^2^) of its surface remained roadless with a median size of 0.2 km^2^ and an average roadless patch size of 0.6 km^2^ (Table 3, Fig. 3b). The median and mean size of Canadian roadless areas were much larger 0.7 and 272 km^2^, respectively. The only roadless area above 100 km^2^ in the European region was the Biebrza National Park in Poland. In the entire Central European study region only 4 areas were larger than 50 km^2^, whereas more than 1,200 were identified in boreal Canada (Table 3). Across the four European countries, over 85% of roadless areas were smaller than 1 km^2^ (Fig. 3b, Table S6).

Visual interpretation of very high-resolution satellite images within the 30 randomly selected roadless areas in the boreal region of Canada (Fig. 3c) revealed a high number of unmapped roads. A total of 34,787 km of additional roads were found to be absent from the OSM dataset in these areas in boreal Canada and required manual mapping. After the visual interpretation and manual road mapping process, the roadless surface within these 30 areas decreased from 127,200 km^2^ to 93,498 km^2^ and their number increased from 30 to 1,408 new roadless areas (Table 3). This represented a loss of 26.5% of the OSM-based roadless surface. One of the 30 roadless areas disappeared completely (Table 3, Table S7, Fig. S3), and only two (below 150 km^2^) were actually roadless and did not change after visual interpretation. The largest loss of roadless surface within a single roadless area was 7597 km^2^, representing 23% of the total initial surface of that patch and resulting in 253 new roadless patches (Fig. S3, ID 21 in Table S7). Most of the manually mapped roads were forest roads with surrounding logging scars. The mean size of the 30 roadless areas decreased from 4223 km^2^ (± s.d. 6531.5 km^2^) to 58 km^2^ (± s.d. 644.2 km^2^). Fifty-nine percent of the newly mapped roadless areas (N = 833) were smaller than 1 km^2^. Two-thirds of the 30 randomly selected roadless areas were in forests, 20% in wetlands, and the remaining 13% in shrubland and herbaceous landscapes (Table S7). After the visual interpretation and road mapping in temperate Central Europe, an additional 257 km of roads were mapped within the 120 selected roadless areas (30 per country, Fig. 3d), which represented a total loss of OSM roadless surface of 164 km^2^ (Table 3). Notably, this led to the complete disappearance of 20 OSM roadless areas while generating 11 new, smaller roadless areas (Table 3). Most newly mapped roads were located in Poland, while the fewest number was manually mapped in Czechia (Tables S8–S11). In Hungary and Czechia most roadless areas were within agricultural fields, whereas in Poland and Slovakia, the selected areas covered more diverse land cover types (Tables S8–S11). Interestingly, the effects of visual interpretation and manual road mapping in Central Europe and Canada, while occurring on vastly different scales and under different road densities, exhibits a remarkable similarity in terms of the proportion of area lost. In Central Europe, the added unmapped roads represented a loss of 28% of the original roadless surface, very similar to the 27% obtained for Canada (Table 3).

## Discussion

Our study revealed considerable shortcomings in the mapping of roadless areas with OSM road data, particularly in remote and relatively intact ecosystems. We proposed two combined methods to provide a comprehensive perspective on the status of roadless areas and road mapping completeness in two contrasting study regions using OSM and high-resolution satellite images. On the one hand, the visual interpretation of random circular plots across the study regions provided a broad overview and contributed to better understanding of the general patterns of roadlessness while offering an objective assessment of road mapping quality in relation to road density and Human Footprint Index. On the other hand, the detailed analysis of the randomly selected roadless areas allowed for a more focused and in-depth examination, revealing a similar reduction of OSM roadless surface (27% and 28%) when unmapped roads were manually included. Up to date, OSM is the most complete, freely available road dataset at a global scale^3,33^. The deficiencies in OSM road mapping were more pronounced in regions with low anthropogenic impact, and therefore, with the greatest potential to contain roadless areas of considerable size that represent functional ecosystems, and where their proper identification and avoidance of further fragmentation would be of high conservation concern^3^.

Our results showed that the challenges of road mapping completeness, including factors such as user interest and the speed of road construction, are particularly pronounced in remote regions with low human influence, like boreal Canada. The quality of OSM road data largely varies due to differences in mapping accuracy and completeness across regions and user contributions^39^. Smaller OSM communities in certain areas result in fewer updates and additions to the road database, particularly in remote or relatively intact regions^35,40^. Infrequent updates and the use of diverse mapping tools by contributors add to inconsistencies in data representation, impacting the overall accuracy and completeness^41^. While OSM remains the most complete open-source global road dataset in terms of road length, its data coverage still tends to concentrate around larger cities, and mapping accuracy decreases with increasing distance from urban areas^40,42^, as found in our study. The absence of roads and inaccurate road mapping in the Canadian OSM road dataset have already been acknowledged by other studies^34,35,43,44^. While Jacobs and Mitchell^43^ and Zhang and Malczewski^34^ focused their quality assessment on a very small scale, specifically on the accuracy of road segments within cities, Zhang and Malczewski^44^ compared OSM road data to a proprietary dataset. Poley et al.^35^ examined the completeness of Canadian road datasets and found that regional (provincial) datasets provided the most complete representation of roadless areas in five provinces, covering nearly 4.1 million km^2^ of Canada. Although freely available regional datasets provided better coverage, the study found that OSM road data was the best alternative when regional datasets were not available (Table S1). The analysis highlighted the difficulty of accurately mapping roadless areas, especially in less developed regions, and emphasized the limitations of global and national road network that underestimated the actual extent of roads in Canada^35^.

Most studies assessing the accuracy and completeness of OSM data are concentrated in Europe, reflecting the substantial user base in that region^34^. In temperate Central Europe, despite a more established mapping community and higher road density and anthropogenic impacts, unmapped roads were still present, albeit in smaller numbers compared to boreal Canada, showing that even in areas almost devoid of roadless areas the pressure on the remaining unfragmented areas persists. Of the randomly selected roadless areas for road mapping, 93% of boreal Canada and 35% of Central Europe had unmapped roads, highlighting the differences in completeness of mapping between the two regions. While 70% of the circular plots in Canada were actually free of roads, 85% of the total area was identified as roadless following the method by Ibisch et al.^3^ with OSM road data. This discrepancy suggests a possible overestimation of roadlessness in the OSM dataset for boreal Canada, indicating considerable incompleteness. In Central Europe, our findings revealed a much higher mapping completeness. The circular plots showed no road-free plots, and the total roadless area surface accounted for only 0.4%. The 15% disparity between the roadless surface and the proportion of road-free plots in boreal Canada, compared to a mere 0.4% difference in Central Europe, provides valuable insights into roadless areas identification and suggest that in some regions, the roadless identification used by us can be quite accurate. We propose that this method should be complemented by an assessment of OSM completeness using the circular plots. Within the OSM road data, predominantly logging roads were underrepresented in the boreal region of Canada, an issue that seems to be common in other forested regions of conservation interest, and which raises concerns due to their overall negative ecological effects^12,45,46^.

Road mapping completeness was notably influenced by anthropogenic factors, with the highest values for road density and the Human Footprint Index observed in plots with both completely and partially mapped roads. The effects of travel time to major cities and the Human Modification Index varied by region, with a stronger influence in boreal Canada. Areas with high anthropogenic impact have generally more populated and developed regions which can lead to a higher road mapping effort and, thus, completeness^33^. This raises questions about the factors influencing road mapping in remote regions and highlights the need to capture local and logging roads, which are critical for ecosystem change and subsequent alteration of biodiversity and ecological processes^46,47^. Visual interpretation allowed us to identify contagious development processes in the boreal region of Canada, where the construction of one road triggers building of new roads and further development^5^. Object-based classification with LiDAR has also proven to be a very effective way to detect logging and gravel roads on a small scale which can be later extrapolated to larger scales^48,49^.

The dynamic growth of the road network, with its continuous construction, modification, and expansion, poses an important challenge for road mapping. Limited financial, technical, and human resources affect the ability to comprehensively map and update road data on regional and global scales^50^. To illustrate it, mapping almost 35,000 km of roads in this study required over 200 working hours. Manual road mapping is highly demanding in terms of human resources, relies on subjective data, can be challenging to interpret, e.g. satellite images, and depends on the competence and accuracy of the cartographer. Deep learning-based techniques, such as convolutional neural networks, have shown promising results in updating road maps and detecting missing roads^29,51^. However, the availability of accurate road data and classifications as training data remains crucial for the effectiveness of these algorithms^27,52^. Although our study relied on manual road mapping, it provided a training dataset of approximately 35,000 km of roads, which can be used for automated road detection and can contribute to improving the accuracy and completeness of road data. Automated road detection methods, coupled with up-to-date satellite images and powerful data processing capabilities, have the potential to enhance road mapping also at a global scale, considering various road types and regions^53,54^. Particularly in remote regions, training an artificial intelligence network capable of detecting logging roads would be highly beneficial, not only to quickly discover illegal logging, but also to prevent the disappearance of valuable roadless ecosystems^28,29,55^. To our knowledge, training of artificial intelligence networks to identify unpaved roads has been done in deserts and in the Brazilian Amazon with promising results^28,29^. Looking ahead, the establishment of a platform and community similar to the Humanitarian OpenStreetMap Team (HOT), but tailored for ecological purposes specifically focused on road mapping in biodiversity-rich regions, would be a valuable initiative.

In both regions, we observed a much lower extent of roadless areas compared to the estimates based on 2013 data by Ibisch et al.^3^, a finding corroborated by several studies^35,56,57^. This may indicate not only better map completeness in the last years, but also the real disappearance of roadless areas due to increasing road construction, even in the highly modified Central Europe. The challenge of accurately assessing the extent of roadless areas is greatest in remote regions, as OSM road mapping is mostly incomplete and these regions are usually subject to uncontrolled and intensive resource extraction which is channeled through roads, leading to irreversible, time-lagged and complex detrimental impacts on ecosystems^9^. Especially in pristine and natural areas severely threatened by the expansion of the road network, an automated system for real-time detection and mapping of roads is urgently needed (Laurance 2018). The impacts of road construction and usage in such areas have severe consequences for biodiversity and ecosystem integrity^11^, can create negative cascading effect, leading to subsequent degradation^58,59^.

This continuous road development highlights the importance of having accurately mapped roads to know where the remaining roadless areas are and to proactively protect them as well as consider them in transport planning avoiding their dissection^3,5,60^. Pristine, unfragmented roadless areas serve as vital strongholds for biodiversity, acting as refuges for numerous species^20^ and are proxies for functional ecosystems, especially forests^23^. It is imperative that these areas are properly identified and road construction banned within them, as a way of protecting them de facto^3,5,17,58^. Such initiatives are possible, even in Europe^20^. Our study highlights the significant challenges and limitations associated with mapping roadless areas, particularly in remote and undisturbed ecosystems, using OSM road data. Here, we introduced a combined approach designed to provide a nuanced view of roadless areas and the extent of road mapping across diverse landscapes. We found notable differences in mapping precision and completeness, with the greatest deficiencies observed in regions with low human impact, such as boreal Canada.

## Conclusion

Our findings emphasize the importance of enhancing roadless area mapping, while acknowledging existing methodological constraints. The combination of up-to-date visual interpretation of random plots and selected roadless areas can provide a reliable assessment of the accuracy of the roadless areas identified. Additionally, enhancing road mapping with deep learning techniques and integrating national or proprietary road data into freely available datasets will substantially improve mapping quality. These advances are crucial to understand the benefits of unfragmented lands and to quantify their contributions to mitigating climate change and preserving functioning ecosystems and biodiversity.

## Materials and methods

### Study areas

**Figure 4:**
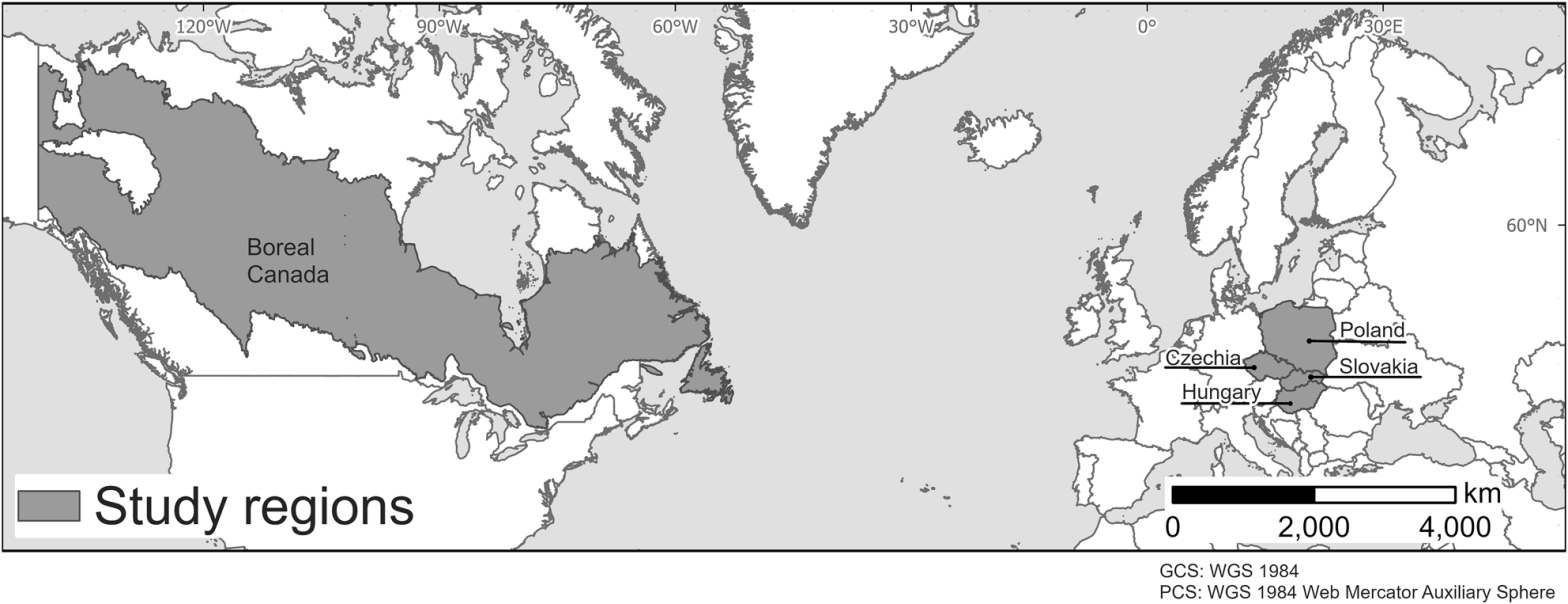
Study regions: boreal region of Canada and temperate Central Europe, including Poland, Slovakia, Czechia, and Hungary. This figure was created using ArcGIS Pro 3.2 (https://www.esri.com/en-us/arcgis/products/arcgis-pro/overview).

The study areas were located in two regions with a priori contrasting road density: the boreal region of Canada and a region in temperate Central Europe, comprising four countries: Poland, Slovakia, Czechia, and Hungary (Fig. 4). The temperate region in Central Europe is regarded as a landscape heavily modified by humans, whereas the Canadian boreal region, and particularly the boreal forests, holds few signs of human modification^61,62^.

Canada is the second largest country in the world, with a relatively low population density of 4.2 people per km^2^ and an area of 9.99 million km^2^, of which 5.4 million km^2^ are within the boreal region^63^. Approximately 50% of the boreal region is covered with forests, which were primarily shaped by natural disturbances such as winds, fires, and insect outbreaks^64^. However, these forests are facing increasing risk from industrial activities, deforestation, and climate change, which is resulting in an increasing number of wildfires and rising temperatures^65^. In contrast, the study region in temperate Central Europe has a much higher population density of more than 100 people per km^2^^66^. It has a surface area of ~ 533,000 km^2^, which corresponds to approximately 10% of the study region in boreal Canada. About 35% of the Central European study region is covered with forests, while up to 80% of the land consists of infrastructure, settlements, and production systems, including agriculture and forestry. As a result, the pressure on the remaining biodiversity and ecosystems is relatively high due to intensive agriculture, transport infrastructure, urban sprawl, deforestation, and climate change-related impacts such as droughts, water scarcity, and floods^67^. Hence, both regions differed in their level of road fragmentation and, therefore, of human footprint^68^.

The Terrestrial Ecoregions dataset was used to delineate the study areas. These data layers were modified by The Nature Conservancy for use in biodiversity planning as part of the process known as "Ecoregional Assessments"^69^. We selected and exported the attribute field ‘Boreal forests/Taiga’ for Canada and for temperate Central Europe, we used the entire country surface which are part of the ‘Temperate Broadleaf and Mixed Forests’ ecoregion. For the boreal region of Canada, land cover data were extracted from the 2015 ‘Land Cover of Canada’ dataset^70^. For temperate Europe, land cover data were extracted from the Copernicus Land Monitoring Service^71^.

### Evaluation of OSM road data completeness

OSM is an inclusive citizen science platform that enables volunteers to collaboratively create, use, and continuously update geographic information^72^ and is regarded as the most complete road dataset in terms of road length on a global scale^3,73^. To comprehensively assess the scope of existing road data, we conducted a comparative analysis involving multiple datasets (Table S1) alongside OSM road data from the year 2020. This initial comparison was done with the Global Roads Inventory Project and a regional Canadian and European road dataset (Table S1). It was confirmed that the total length of mapped roads in the OSM road dataset was the highest in both regions compared to the other available datasets. Road density serves as an indicator of human activity and development and allows for cross-regional comparisons and analysis of human impact on the environment. Higher road density indicates greater fragmentation of the landscape and ecosystems. To assess road-related environmental impacts, such as the degree of road fragmentation, we created a road density raster from the 2020 OSM road dataset. The raster was computed for both study regions by dividing the total road length within a 5 km^2^ grid by its area. This approach highlighted variations in road density between the temperate Central European and the Canadian boreal regions. We hypothesized that regions with higher anthropogenic impact would exhibit better mapping compared to regions with lower human influence. To investigate this hypothesis, we analyzed the relationship of road mapping completeness with human-related variables such as road density, travel time to major cities, Human Footprint Index, and Human Modification Index^32,36–38^. The Human Footprint Index dataset used in this study, which was updated by Venter et al.^37^ contains data for the year 2009, whereas the Human Modification Index, developed by Kennedy et al.^38^ consists of data from 2016. Oaklead and Kennedy^74^ conducted a comparative analysis between the Human Footprint Index and their Human Modification Index, providing valuable insights into the similarities and differences between these two indices.

To assess the completeness of the 2020 OSM road dataset, we randomly selected 1000 cells per country from a 500 × 500 m square grid that fully covered both study regions. Within each random cell, we generated circular buffers with a radius of 1 km around the cell’s centroid, generating circular plots of 3.14 km^2^. The circular plots encompassed 0.06% of the boreal region in Canada (N = 1000 plots), and in the temperate region of Central Europe, they encompassed 0.6% (N = 1000 plots). To address differences among the four European countries, we further randomly selected 1000 plots per country, including the previously 1000 selected, encompassing 2.4% of the study area (N = 4000 plots).

We reviewed the completeness of the OSM road data through visual interpretation of Esri’s high-resolution base map within the circular plots. Visual interpretation of a satellite image consists of analyzing an image recorded by a satellite sensor and interpreting the features and patterns visible on the image. This process involves examining the image at different scales and using visual landmarks to identify and interpret different features on the ground. In this case, roads were identified and mapped as features on the ground. We used the ArcGIS Pro 2.8 World Imagery base map at a scale of 1:30,000 for the visual interpretation. Satellite images from various sources from the period 2010–2020 with a resolution of 0.3–0.5 m were available for the respective sections of the examined base map. Any section that was not available in the time frame of 2019–2020 was later verified in Google Earth Pro and on Sentinel-2 satellite images from the Sentinel-2 hub to ensure that roads or linear infrastructures were still visible in the year 2020. After visual interpretation, each circular plot was classified according to the following categories of road map completeness: plots with all roads completely mapped by OSM, plots with roads partially mapped by OSM, plots where all roads were unmapped by OSM, plots with no roads, and plots containing other linear infrastructures. Linear infrastructures that could not be verified as roads could be powerlines, seismic lines for oil and gas exploration, firebreaks (to prevent wildfires) or other anthropogenic structures. We computed road density, travel time to major cities, Human Footprint Index, and Human Modification Index for each of the 1000 random circular plots in both study regions and for all countries^32,36–38^. The values from each of the aforementioned datasets were extracted for each of the 1000 circular plots for both study regions. Histograms were constructed for each variable to analyze the frequency distribution of data values, providing a visual representation of the spread and concentration of observations within each variable (Fig. S1). To assess the relationships between variables, correlation matrices were computed using Spearman correlation coefficient (Table S3). The mean, and standard deviation of these explanatory variables were then calculated for every plot category.

To assess whether road completeness was associated with the explanatory variables indicating human influence, we initially evaluated if spatial autocorrelation affected the results by fitting a Generalized Least Squares (GLS) model^75^. We represented the categorical completeness index as a continuous variable for use as a response in the model, using values ranging from 1—not mapped to 3—completely mapped. For the analyses, we took a subset of locations where roads were present, excluding the categories ‘no roads’ and ‘other linear infrastructure’. Explanatory variables included road density, travel time to major cities, the Human Footprint Index and the Human Modification Index, and their interaction with country. We accounted for spatial autocorrelation in the data by including a spatial correlation structure in the GLS model. Location coordinates were transformed to equidistant projection (UTM/WGS 84), so that the distances were comparable. Numerical explanatory variables were standardized prior to model fitting to facilitate model convergence. We compared GLS models with alternative spatial correlation structures as well as an ordinary linear regression model (i.e. not accounting for spatial autocorrelation) by ranking the models using the second-order Akaike Information Criterion (AIC_*c*_) and further by examining model coefficients. Next, we applied an ordinal logistic regression model, which allows the use of categorical response where groups have a natural order^76^, to the categorical road completeness index. This model included the same explanatory variables as previously and their interactions with region (Boreal Canada/Central Europe) and country. Alternative models with an additive or multiplicative effect of a country were ranked by AIC_*c*_^77^. We used R packages ‘ordinal’^78^ for ordinal regression and `nlme`^79^ for GLS models.

### Assessing roadlessness

We followed the roadless areas definition by Ibisch et al.^3^, by creating a geodesic buffer of 1 km on each side of every road (Fig. S6). This threshold was chosen as a conservative measure based on an extensive literature review^3^, which found that the most intense, direct, and negative impacts of roads are within 1 km of the road. This buffer is called the road-effect zone and encompasses the surrounding area with significant ecological impacts caused by roads^7^. The roadless areas are therefore defined as areas more than 1 km away from any kind of road, and thus, relatively free of road impacts. We mapped roadless areas for the year 2020 based on OSM road data for both the boreal region of Canada and the temperate region of Central Europe, after delimiting both areas as explained in the previous section. We extracted the number of the resulting roadless areas and their surface for both regions.

Out of the identified roadless areas, we selected 30 per country for verification to confirm the real absence of roads by visual interpretation as described above. The selection process was carried out randomly but aiming for a comprehensive representation of all roadless area sizes. To achieve this, we employed a weighting factor based on the proportion of an individual roadless area's size relative to the total roadless area size for both study regions. This approach prevented a bias towards solely selecting smaller roadless areas, which are more abundant^3^, and allowed for a representation of all available sizes. We visually checked 30 roadless areas for boreal Canada and 30 for each of the temperate Central European countries. In cases where roads were found, we manually mapped the missing road segments. This method of road mapping involved visually identifying, tracing and delineating road features on maps. To ensure accurate and efficient visual interpretation and mapping using the Esri basemap satellite imagery as described above, a scale of 1:30,000 was chosen, balancing the need for detailed mapping, and working effort. Once all identifiable missing roads were mapped within each roadless area, their length was calculated, and they were buffered with a 1 km geodetic buffer and incorporated into the existing roadless area layer. Then, we identified the new roadless areas and calculated their number and size, as well as the total loss of roadless surface after including the unmapped roads (Tables S7–S11, Fig. S5).

All analyses were conducted with ArcGIS Pro 2.9^80^, and R 4.1.3^81^.

## Supplementary Information

Supplementary Information.

## Data Availability

Manually mapped roads are available from the corresponding author on request. Data of circular plots and roadless areas for both study regions are available from the Zenodo repository.
