# Author Correction: Mapping roadless areas in regions with contrasting human footprint

**DOI:** 10.1038/s41598-024-79802-4

**Published:** 2024-11-25

**Authors:** Monika T. Hoffmann, Katarzyna Ostapowicz, Kamil Bartoń, Pierre L. Ibisch, Nuria Selva

**Affiliations:** 1 Institute of Nature Conservation, Polish Academy of Sciences, 31‑120 Krakow, Poland; 2 FRAM-High North Centre for Climate and the Environment, Norwegian Institute of Nature Research (NINA), 9296 Tromsø, Norway; 3 Institute of Geography and Spatial Management, Faculty of Geography and Geology, Jagiellonian University, 30‑387 Krakow, Poland; 4 Centre for Econics and Ecosystem Management, Eberswalde University for Sustainable Development, 16225 Eberswalde, Germany; 5 Departamento de Ciencias Integradas, Facultad de Ciencias Experimentales, Centro de Estudios Avanzados en Física, Matemáticas y Computación, Universidad de Huelva, 21071 Huelva, Spain; 6 Estación Biológica de Doñana, Consejo Superior de Investigaciones Científicas, 41092 Sevilla, Spain

Correction to: *Scientific Reports* 10.1038/s41598-024-55283-3, publisher online 27 February 2024

The original version of this Article contained errors in Fig. 2, where the labels of the road completeness level 1 and 3 were switched. The original Fig. 2 and accompanying legend appear below.

**Fig. 2:**
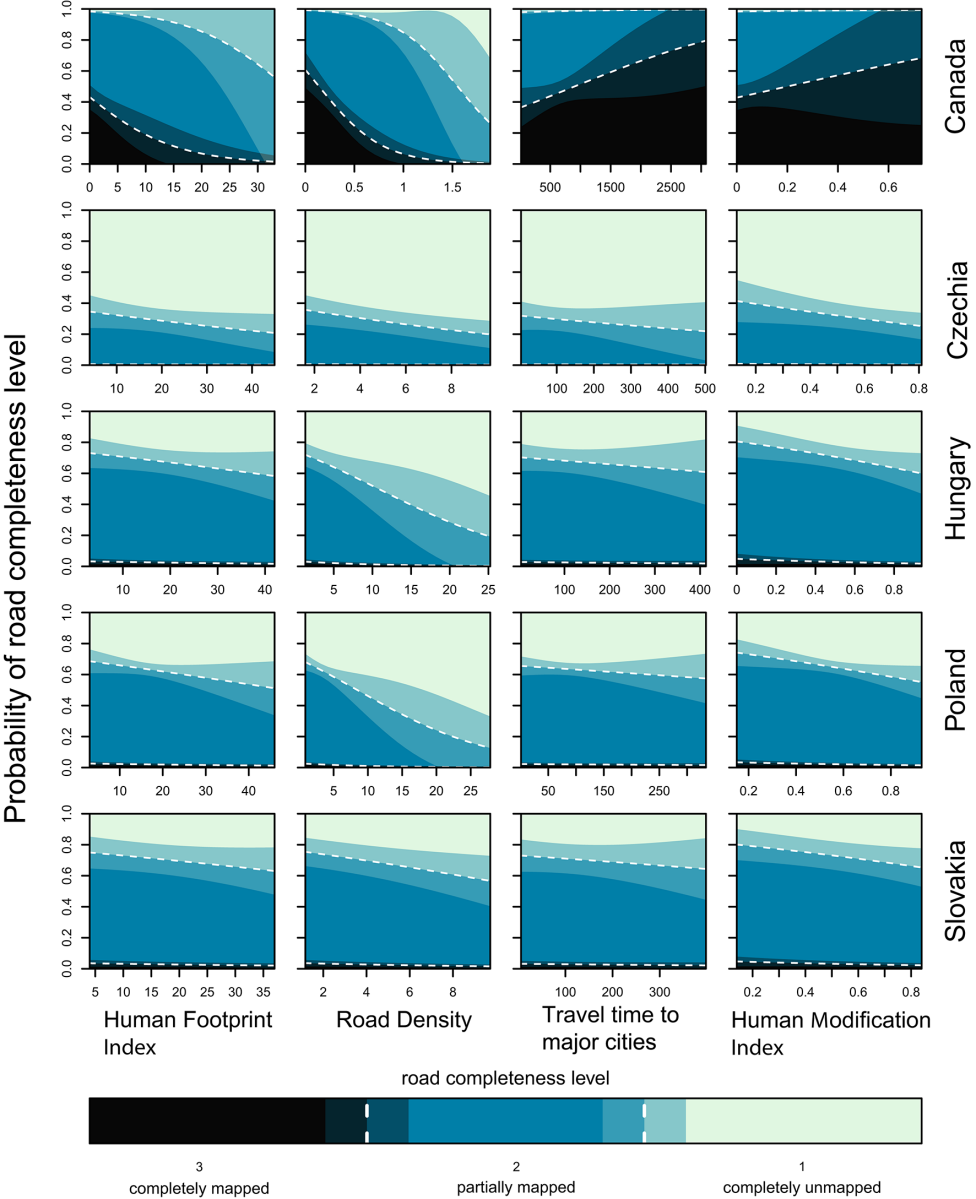
Proportion of plots with three categories of road completeness (completely mapped = 3, partially mapped = 2, completely unmapped = 1), as predicted by the ordinal regression model, in relation to the four variables of anthropogenic influence in each of the five study countries. Shaded regions represent the levels of completeness. Mean values are shown by dashed lines, and intermediate shading indicates 95% confidence intervals.

As a result of this error, Fig. 2 legend,

“Proportion of plots with three categories of road completeness (completely mapped = 3, partially mapped = 2, completely unmapped = 1), as predicted by the ordinal regression model, in relation to the four variables of anthropogenic influence in each of the five study countries. Shaded regions represent the levels of completeness. Mean values are shown by dashed lines, and intermediate shading indicates 95% confidence intervals.”

now reads:

“Proportion of plots with three categories of road completeness (completely mapped = 1, partially mapped = 2, completely unmapped = 3), as predicted by the ordinal regression model, in relation to the four variables of anthropogenic influence in each of the five study countries. Shaded regions represent the levels of completeness. Mean values are shown by dashed lines, and intermediate shading indicates 95% confidence intervals.”

Additionally, Table 2 legend contained the same error,

“Analysis of deviance table (type II tests) for the ordinal regression model of road completeness (completely mapped = 3, partially mapped = 2, completely unmapped = 1).”

now reads:

“Analysis of deviance table (type II tests) for the ordinal regression model of road completeness (completely mapped = 1, partially mapped = 2, completely unmapped = 3).”

The original Article has been corrected.

