# Supplementary material for: Mapping roadless areas in regions with contrasting human footprint
Source: Sci Rep. 2024 Feb 27;14:4722. doi: 10.1038/s41598-024-55283-3 (PMC10899609; doi:10.1038/s41598-024-55283-3)
Supplement: Supplementary file 1 — Supplementary Information. [file 41598_2024_55283_MOESM1_ESM.pdf]

*Scientific Reports*

SUPPLEMENTARY MATERIAL

# Mapping roadless areas in regions with contrasting human footprint

Monika T. Hoffmann[1,2,*], Katarzyna Ostapowicz[3,2], Kamil Bartoń [1], Pierre L. Ibisch[4], and Nuria Selva[1,5,6]

[1] Institute of Nature Conservation, Polish Academy of Sciences, 31-120 Krakow, Poland.
[2] Institute of Geography & Spatial Management, Faculty of Geography & Geology, Jagiellonian University, 30-387 Krakow, Poland.
[3] Norwegian Institute of Nature Research (NINA), FRAM - High North Centre for Climate and the Environment, Tromsø 9296, Norway.
[4] Centre for Econics and Ecosystem Management, Eberswalde University for Sustainable Development, 16225 Eberswalde, Germany.
[5] Departamento de Ciencias Integradas, Facultad de Ciencias Experimentales, Centro de Estudios Avanzados en Física, Matemáticas y Computación, Universidad de Huelva, 21071 Huelva, Spain.
[6] Estación Biológica de Doñana, Consejo Superior de Investigaciones Científicas, 41092 Sevilla, Spain.
*Corresponding author: monika.t.hoffmann@gmail.com

**This PDF file includes:**

Supplementary Materials and Methods
Tables S1-S11
Figs. S1-S6
References

## Supplementary Materials and Methods

### Roadless areas definition and data processing

This study is based on the OpenStreetMap (OSM) road dataset, a dynamic and openly accessible database that is updated daily and allows copying, reproduction, redistribution, and modification with proper attribution to OSM and its contributors [1]. Roads were obtained from OSM for 2020 and included 36 different road categories [2, 3]. All categories have been incorporated into our analysis, given their characteristic of facilitating human and (motorized) access, a factor recognized as a potential threat and risk to various species and ecosystems, as revised by Ibisch et al. [4]. Each road was buffered with a geodesic buffer of 1 km on each side of every road (Figure S5). After creating the buffer around each road, the area of boreal Canada and temperate Central Europe were extracted to obtain a layer containing only roadless areas located 1 km away from the nearest road. 'Natural Earth data' provided the layer for lakes, which were extracted from the country layer to exclude large water bodies from this analysis [5]. Wetlands, smaller lakes, and streams were included in the roadless area assessment.

### Anthropogenic influences and road mapping within the circular plots randomly selected

We hypothesized that regions with higher anthropogenic influences would exhibit better mapping compared to regions with lower anthropogenic impact. To investigate this, we analyzed various datasets related to road mapping completeness, including road density, Travel time to major cities, Human Footprint Index, and Human Modification Index [2, 6, 7, 8]. The distribution of all four explanatory variables and the completeness of roads was examined and a correlation matrix was created (Fig. S1, Table S3). We then examined only circular plots containing roads (mapped and unmapped) for statistical testing (Tables S4, S5).

### Forest cover and roadless areas

The Canadian boreal region comprises approximately 2.2 million km² of forest, with 1.4 million km² designated as forested roadless areas [9]. Conversely, Central Europe's forest cover spans 187,447 km², as per Copernicus data, with a mere 821 km² representing forested roadless areas [10]. In both regions the forest cover within the 30 selected roadless

areas was calculated before and after road mapping. In both regions a loss of 40% of roadless forest cover was detected.

**Table S1.** Road network length of the two study regions according to different road datasets. The references to the dataset links are provided in the reference list.

| Road dataset | Boreal Canada | Central Europe | Dataset links |
|---|---|---|---|
| CIESIN gROADS, v1. 2013 [11] | 52,177 km | 62,782 km | https://sedac.ciesin.columbia.edu/data/set/groads-global-roads-open-access-v1 |
| GRIP 2018 [12] | 63,365 km | 403,457 km | https://www.globio.info/download-grip-dataset |
| OSM 2020 [2] | 528,127 km | 1,761,143 km | https://download.geofabrik.de/ |
| Canadian National Road Network (NRN) 2020 [13] | 319,245 km | | https://canadiangis.com/national-road-network-nrn-canadian-open-data.php |
| EuroGeographics [14] | | 115,738 km | https://public.opendatasoft.com/explore/dataset/europe-road/export/?refine.icc=BE |

**Table S2**. Summary of the visual interpretation of the randomly selected circular plots (n = 1000 per country, 3.14 km² each) for each of the Central European countries. It shows the number of circular plots within the following categories: plots with all roads completely mapped, plots with roads partially mapped, plots with all roads unmapped, and plots without roads.

| Country | Plot categories | No. of plots |
|---|---|---|
| Poland | Completely mapped roads | 639 |
| | Partially mapped roads | 355 |
| | Unmapped roads | 1 |
| | No roads | 5 |
| Slovakia | Completely mapped roads | 523 |
| | Partially mapped roads | 473 |
| | Unmapped roads | 4 |
| Czechia | Completely mapped roads | 713 |
| | Partially mapped roads | 286 |
| | No roads | 1 |
| Hungary | Completely mapped roads | 657 |
| | Partially mapped roads | 335 |
| | Unmapped roads | 3 |
| | No roads | 5 |

**Table S3.** Correlation matrix between variables of human influences in the 1000 randomly selected plots in Central Europe and boreal Canada. Correlation coefficients (Spearman) range from -1 to 1.

| **Central Europe** | Road completeness | Road density | Travel time to major cities | Human Footprint Index | Human Modification Index |
|---|---|---|---|---|---|
| Road completeness | | | | | |
| Road density | -0.16 | | | | |
| Travel time to major cities | 0.10 | -0.63 | | | |
| Human Footprint Index | -0.16 | 0.65 | -0.35 | | |
| Human Modification Index | -0.23 | 0.39 | -0.36 | 0.18 | |

| **Boreal Canada** | Road completeness | Road density | Travel time to major cities | Human Footprint Index | Human Modification Index |
|---|---|---|---|---|---|
| Road completeness | | | | | |
| Road density | -0.64 | | | | |
| Travel time to major cities | 0.60 | -0.82 | | | |
| Human Footprint Index | -0.46 | 0.43 | -0.3 | | |
| Human Modification Index | -0.66 | 0.59 | -0.55 | 0.54 | |

**Table S4.** Model selection table and coefficient values of the Generalized Least Squares models. Models ranked by $AIC_c$. The response variable is road completeness (fully mapped = 3, partially mapped = 2, not mapped = 1) and the explanatory variables are road density ($km/km^2$), travel time to major cities (min), Human Footprint Index (ranging from 0 to 50, low values indicated low human footprint) and Human Modification Index (ranging from 0 to 1, low values indicated low degree of landscape modification by humans).

| (Intercept) | Human Modification Index | Human Footprint Index | Road density | Travel time to major cities | Correlation | df | Delta $AIC_c$ | Akaike weight |
|---|---|---|---|---|---|---|---|---|
| 3.47 | 0.24 | -0.08 | 1.49 | -0.04 | Exponential | 28 | 0 | 0.47 |
| 3.47 | 0.24 | -0.08 | 1.48 | -0.04 | Rational | 28 | 0.15 | 0.44 |
| 3.90 | 0.22 | -0.08 | 1.67 | -0.04 | Spherical | 28 | 3.3 | 0.09 |
| 3.46 | 0.24 | -0.09 | 1.55 | -0.05 | Gaussian | 28 | 20.4 | 0 |
| 3.47 | 0.24 | -0.09 | 1.57 | -0.05 | None | 26 | 21.76 | 0 |
| 3.47 | 0.24 | -0.09 | 1.57 | -0.05 | Linear | 28 | 25.94 | 0 |

**Table S5**. Model selection table of the Ordinal Regression models accounting for country differences in Europe in relation to road completeness**.** Models ranked by $AIC_c$.

| | df | Delta $AIC_c$ | Akaike weight |
|---|---|---|---|
| country effect additive | 14 | 0 | 0.58 |
| country effect none | 26 | 0.61 | 0.42 |
| country effect multiplicative | 11 | 48.83 | 0 |

**Table S6.** Extent and amount of roadless areas (calculated by creating a 1km geodesic buffer around each road and extracting the remaining area) across boreal Canada and each of the selected Central European countries. The table provides information on the number of roadless areas in different size classes, along with the total roadless surface**.**

| Country | Size of the roadless area in km² | No. roadless areas | Total roadless surface in km² |
|---|---|---|---|
| Boreal Canada | (0, 1] | 9,112 | 2,051 |
| | (1, 2] | 1,784 | 2,604 |
| | (2, 4] | 1,494 | 4,325 |
| | (4, 10] | 1,484 | 9,576 |
| | (10, 20] | 832 | 11,802 |
| | (20, 50] | 841 | 26,803 |
| | (50, 100] | 418 | 29,822 |
| | (100, 250] | 357 | 55,743 |
| | (250, 500] | 173 | 61,332 |
| | (500, 1,000] | 130 | 93,597 |
| | (1,000, 10,000] | 131 | 386,848 |
| | (10,000, 100,000] | 23 | 733,672 |
| | (100,000,1,000,000] | 6 | 1,968,548 |
| | (1,000,000, 1,173,890] | 1 | 1,173,889 |
| | Total | 16,786 | 4,560,608.4 |
| Poland | (0, 1] | 1,781 | 288 |
| | (1, 2] | 128 | 177 |
| | (2, 4] | 73 | 202 |
| | (4, 10] | 34 | 200 |
| | (10, 20] | 7 | 81 |
| | (20, 50] | 2 | 57 |
| | (50, 100] | 2 | 143 |
| | (100, 250] | 1 | 107 |
| | Total | 2,028 | 1,256 |
| Slovakia | (0, 1] | 375 | 65 |
| | (1, 2] | 35 | 47 |
| | (2, 4] | 14 | 38 |
| | (4, 10] | 3 | 20 |
| | (10, 20] | 1 | 12 |
| | Total | 428 | 182 |
| Czechia | (0, 1] | 124 | 16 |
| | (1, 2] | 9 | 12 |
| | (2, 4] | 7 | 21 |
| | (4, 10] | 2 | 12 |
| | (10, 20] | 1 | 13 |
| | Total | 143 | 7 |
| Hungary | (0, 1] | 782 | 141 |

| | | |
|---|---|---|
| (1, 2] | 74 | 108 |
| (2, 4] | 41 | 109 |
| (4, 10] | 22 | 127 |
| (10, 20] | 3 | 37 |
| (20, 50] | 2 | 74 |
| (50, 100] | 1 | 53 |
| Total | 925 | 649 |

**Table S7.** Outcome of the visual interpretation of 30 randomly selected roadless areas in boreal Canada, showing the assigned ID, the surface of the largest roadless area after road mapping, the sum of all roadless areas created after road mapping, the reduction in size of roadless areas after road mapping, the median size of the newly identified areas after road mapping and the main landcover type.

| ID | No. of newly identified roadless areas after road mapping | Roadless area surface before road mapping ($km^2$) | Roadless area surface after road mapping (largest patch, $km^2$) | Total surface of mapped roadless areas (sum of all new roadless areas after road mapping, $km^2$) | Reduction of roadless area surface after road mapping ($km^2$) | Reduction of roadless area surface after mapping (%) | Median size of roadless areas after road mapping ($km^2$) | Landcover type |
|---|---|---|---|---|---|---|---|---|
| 1 | 2 | 25.2 | 23.5 | 23.5 | 1.7 | 0.1 | 11.7 | Forest |
| 2 | 19 | 526.7 | 129.8 | 301.4 | 225.3 | 42.8 | 15.9 | Forest |
| 3 | 26 | 456 | 119.6 | 262.6 | 193.4 | 42.4 | 0.3 | Herbs |
| 4 | 1 | 1.0 | 1.018 | 1.0 | 0 | 0 | - | Forest |
| 5 | 33 | 504.1 | 13.3 | 37.6 | 466.5 | 92.5 | 0.2 | Forest |
| 6 | 1 | 760.9 | 720.1 | 720.09 | 40.8 | 5.4 | - | Forest |
| 7 | 8 | 1,989.6 | 1,791.9 | 1,819.2 | 170.4 | 8.6 | 2.5 | Forest |
| 8 | 103 | 6,738.6 | 1,804.5 | 4,546.2 | 2,192.4 | 32.5 | 0.4 | Wetland |
| 9 | 0 | 13.5 | 0 | 0 | 13.5 | 100 | - | Forest |
| 10 | 11 | 541.0 | 221.2 | 276 | 265.0 | 49 | 0.5 | Forest |
| 11 | 112 | 4,437 | 397.8 | 2,023.2 | 2,413.8 | 54.4 | 1.0 | Forest |
| 12 | 7 | 215.8 | 92.5 | 117.1 | 98.7 | 45.7 | 1.7 | Forest |
| 13 | 150 | 5,329.4 | 513.9 | 2,367.8 | 2,961.6 | 55.6 | 0.7 | Forest |
| 14 | 38 | 7,190.5 | 6,320.8 | 6,466.7 | 723.8 | 10.1 | 0.2 | Wetland |
| 15 | 31 | 943.2 | 136.4 | 479.9 | 463.3 | 49.1 | 1.1 | Wetland |
| 16 | 12 | 328.1 | 92.6 | 113.3 | 214.8 | 65.5 | 0.7 | Herbs |
| 17 | 29 | 13,776.5 | 11,346.2 | 12,947.7 | 828.8 | 6.0 | 0.8 | Forest |

| | | | | | | | | |
|---|---|---|---|---|---|---|---|---|
| 18 | 16 | 1,234.1 | 839.4 | 887.7 | 346.4 | 28.1 | 0.4 | Forest |
| 19 | 115 | 4,115.6 | 432.3 | 1,721.4 | 2,394.2 | 58.3 | 0.8 | Forest |
| 20 | 1 | 55.0 | 55.0 | 55.0 | 0 | 0 | - | Forest |
| 21 | 253 | 32,504.6 | 16,302.6 | 24,907.2 | 7,597.4 | 23.4 | 0.3 | Forest |
| 22 | 7 | 9,364.7 | 9,181.7 | 9,246.5 | 118.2 | 1.3 | 0.3 | Forest |
| 23 | 7 | 2,614.9 | 2,448.2 | 2,506.2 | 108.7 | 4.2 | 2.2 | Forest |
| 24 | 2 | 6,463.9 | 6,408.1 | 6,408.0 | 55.9 | 0.9 | 3.2 | Shrubland |
| 25 | 211 | 5,655.9 | 130.6 | 475.6 | 5,180.3 | 91.6 | 0.3 | Wetland |
| 26 | 21 | 1,236.5 | 5 | 17.4 | 1,219.1 | 98.6 | 0.1 | Wetland |
| 27 | 78 | 3,508.6 | 358.1 | 1,321.7 | 2,186.9 | 62.3 | 0.6 | Shrubland |
| 28 | 112 | 3,980.2 | 262.9 | 927.3 | 3,052.9 | 76.7 | 0.6 | Forest |
| 29 | 1 | 125.2 | 125.2 | 125.2 | 0 | 0 | - | Forest |
| 30 | 1 | 12,563.3 | 12,395.0 | 12,395.0 | 168.3 | 1.3 | - | Forest |
| | 1408 | 127,199.5 | | 93,497.6 | 33,701.9 | 26.5 | | |

**Table S8.** Outcome of the visual interpretation of 30 randomly selected roadless areas in Poland, showing the assigned ID, the surface of the largest roadless area after road mapping, the sum of all roadless areas created after road mapping, the reduction in size of roadless areas after road mapping, and the main landcover type.

| ID | No. of newly identified roadless areas after road mapping | Roadless area surface before road mapping in ($km^2$) | Roadless area surface after road mapping (largest patch, $km^2$) | Total surface of mapped roadless areas (sum of all new roadless areas after road mapping, $km^2$) | Reduction of roadless area surface after road mapping ($km^2$) | Reduction of roadless area surface after mapping (%) | Landcover type |
|---|---|---|---|---|---|---|---|
| 1 | 0 | 0.03 | 0 | 0.03 | 0 | 0 | Agriculture |
| 2 | 0 | 0.1 | 0 | 0.1 | 0 | 0 | Lake |
| 3 | 0 | 0.1 | 0 | 0.1 | 0 | 0 | Agriculture |
| 4 | 0 | 0.2 | 0 | 0.2 | 0 | 0 | Agriculture |
| 5 | 0 | 0.3 | 0 | 0.3 | 0 | 0 | Forest |
| 6 | 0 | 0.6 | 0 | 0.6 | 0 | 0 | Forest |
| 7 | 0 | 0.6 | 0 | 0.6 | 0 | 0 | Grassland |
| 8 | -1 | 0.6 | 0 | 0 | 0.6 | 100 | Forest |
| 9 | 0 | 0.8 | 0 | 0.8 | 0 | 0 | Forest |
| 10 | -1 | 1.2 | 0 | 0 | 1.2 | 100 | Forest |
| 11 | 0 | 1.7 | 0 | 1.7 | 0 | 0 | Forest |
| 12 | 0 | 1.8 | 0 | 1.8 | 0 | 0 | Forest |
| 13 | 0 | 2.6 | 0 | 2.0 | 0.6 | 24.1 | Forest |
| 14 | 0 | 2.7 | 0 | 2.7 | 0 | 0 | Lake |
| 15 | 0 | 2.8 | 0 | 2.1 | 0.7 | 24.3 | Wetland |
| 16 | 2 | 3.9 | 0.3 | 0.5 | 3.4 | 87.2 | Grassland |
| 17 | 0 | 3.9 | 0 | 3.9 | 0 | 0 | Forest |
| 18 | 0 | 3.9 | 0 | 3.9 | 0 | 0 | Lake |
| 19 | -1 | 4.3 | 0 | 0 | 4.3 | 100 | Forest |

| | | | | | | | |
|---|---|---|---|---|---|---|---|
| 20 | -1 | 4.5 | 0 | 0 | 4.5 | 100 | Forest |
| 21 | -1 | 4.7 | 0 | 0 | 4.7 | 100 | Forest |
| 22 | 0 | 5.7 | 0 | 5.7 | 0 | 0 | Lake |
| 23 | 2 | 8.2 | 0.3 | 0.5 | 7.7 | 93.7 | Forest |
| 24 | 0 | 9.0 | 0 | 1.5 | 7.5 | 83.1 | Forest |
| 25 | 0 | 10.8 | 0 | 10.8 | 0 | 0 | Lake |
| 26 | -1 | 13.1 | 0 | 0 | 13.1 | 100 | Forest |
| 27 | 0 | 21.8 | 0 | 21.8 | 0 | 0 | Forest |
| 28 | 0 | 61.6 | 0 | 61.6 | 0 | 0 | Lake |
| 29 | 2 | 81.5 | 76.6 | 76.7 | 4.8 | 5.9 | Agriculture |
| 30 | 2 | 106.8 | 61.2 | 96.2 | 10.6 | 9.9 | Grassland |
| Total | 0 | 359.7 | | 295.9 | 63.7 | | |

**Table S9.** Outcome of the visual interpretation of 30 randomly selected roadless areas in Slovakia, showing the assigned ID, the surface of the largest roadless area after road mapping, the sum of all roadless areas created after road mapping, the reduction in size of roadless areas after road mapping, and the main landcover type.

| ID | No. of newly identified roadless areas after road mapping | Roadless area surface before road mapping in ($km^2$) | Roadless area surface after road mapping (largest patch, $km^2$) | Total surface of mapped roadless areas (sum of all new roadless areas after road mapping, $km^2$) | Reduction of roadless area surface after road mapping ($km^2$) | Reduction of roadless area surface after mapping (%) | Landcover type |
|---|---|---|---|---|---|---|---|
| 1 | 0 | 1.8 | 0 | 0 | 1.8 | 99.2 | Agriculture |
| 2 | -1 | 0.4 | 0 | 0 | 0.4 | 100 | Agriculture |
| 3 | 0 | 0 | 0 | 0 | 0 | 0 | Agriculture |
| 4 | 0 | 0 | 0 | 0 | 0 | 0 | Forest |
| 5 | 0 | 0 | 0 | 0 | 0 | 0 | Agriculture |
| 6 | -1 | 0.7 | 0 | 0 | 0.7 | 100 | Agriculture |
| 7 | 0 | 0.1 | 0 | 0.1 | 0 | 0 | Agriculture |
| 8 | 0 | 0 | 0 | 0 | 0 | 0 | Agriculture |
| 9 | 0 | 0.1 | 0 | 0.1 | 0 | 0 | Forest |
| 10 | 0 | 1.0 | 0 | 0.2 | 0.7 | 75.8 | Agriculture |
| 11 | 0 | 0.7 | 0 | 0.7 | 0 | 0 | Agriculture |
| 12 | 0 | 0 | 0 | 0 | 0 | 0 | Forest |
| 13 | 0 | 0 | 0 | 0 | 0 | 0 | Agriculture |
| 14 | 0 | 0 | 0 | 0 | 0 | 0 | Forest |
| 15 | 0 | 0 | 0 | 0 | 0 | 0 | Forest |
| 16 | 0 | 0 | 0 | 0 | 0 | 0 | Agriculture |
| 17 | 2 | 2.0 | 0.2 | 0.3 | 1.7 | 86.2 | Forest |
| 18 | 0 | 0 | 0 | 0 | 0 | 0 | Forest |
| 19 | 0 | 1.6 | 0 | 0.1 | 1.5 | 95.8 | Forest |

| | | | | | | | |
|---|---|---|---|---|---|---|---|
| 20 | 0 | 0 | 0 | 0 | 0 | 0 | Mountain |
| 21 | 0 | 0.4 | 0 | 0.4 | 0 | 0 | Agriculture |
| 22 | 0 | 0.4 | 0 | 0.4 | 0 | 0 | Agriculture |
| 23 | -1 | 0.5 | 0 | 0 | 0.5 | 100 | Forest |
| 24 | 0 | 0 | 0 | 0 | 0 | 0 | Forest |
| 25 | 0 | 0.1 | 0 | 0.1 | 0 | 0 | Forest |
| 26 | 0 | 0 | 0 | 0 | 0 | 0 | Forest |
| 27 | 0 | 0.1 | 0 | 0.1 | 0 | 0 | Forest |
| 28 | 0 | 0.1 | 0 | 0 | 0 | 0 | Forest |
| 29 | 0 | 2.1 | 0 | 2.1 | 0 | 0 | Lake |
| 30 | 0 | 0.5 | 0 | 0.5 | 0 | 0 | Forest |
| Total | | 12.6 | | 5.2 | 7.3 | | |

**Table S10.** Outcome of the visual interpretation of 30 randomly selected roadless areas in Czechia, showing the assigned ID, the sum of all roadless areas created after road mapping, the reduction in size of roadless areas after road mapping, and the main landcover type.

| ID | No. of newly identified roadless areas after road mapping | Roadless area surface before road mapping in ($km^2$) | Total surface of mapped roadless areas (sum of all new roadless areas after road mapping, $km^2$) | Reduction of roadless area surface after road mapping ($km^2$) | Reduction of roadless area surface after mapping (%) | Landcover type |
|---|---|---|---|---|---|---|
| 1 | 0 | 0.4 | 0.4 | 0 | 0 | Agriculture |
| 2 | 0 | 0 | 0 | 0 | 0 | Agriculture |
| 3 | 0 | 0.1 | 0.1 | 0 | 0 | Agriculture |
| 4 | 0 | 0.9 | 0.9 | 0 | 0 | Agriculture |
| 5 | -1 | 0.3 | 0 | 0.3 | 100 | Agriculture |
| 6 | 0 | 1.7 | 1.7 | 0 | 0 | Agriculture |
| 7 | -1 | 0.3 | 0 | 0.3 | 100 | Agriculture |
| 8 | 0 | 0.2 | 0.2 | 0 | 0 | Agriculture |
| 9 | -1 | 0.2 | 0 | 0.2 | 100 | Agriculture |
| 10 | 0 | 0 | 0 | 0 | 0 | Agriculture |
| 11 | 0 | 0 | 0 | 0 | 0 | Agriculture |
| 12 | 0 | 0.1 | 0.1 | 0 | 0 | Forest |
| 13 | 0 | 0 | 0 | 0 | 0 | Grassland |
| 14 | 0 | 0.1 | 0.1 | 0 | 0 | Forest |
| 15 | 0 | 0 | 0 | 0 | 0 | Agriculture |
| 16 | 0 | 0.1 | 0.1 | 0 | 0 | Grassland |
| 17 | 0 | 0.1 | 0.1 | 0 | 0 | Grassland |
| 18 | 0 | 0.1 | 0.1 | 0 | 0 | Grassland |
| 19 | 0 | 0 | 0 | 0 | 0 | Forest |
| 20 | -1 | 0 | 0 | 0 | 100 | Forest |
| 21 | 0 | 0 | 0 | 0 | 0 | Forest |
| 22 | 0 | 0 | 0 | 0 | 0 | Agriculture |

| | | | | | | |
|---|---|---|---|---|---|---|
| 23 | 0 | 0 | 0 | 0 | 0 | Forest |
| 24 | 0 | 0 | 0 | 0 | 0 | Forest |
| 25 | 0 | 0.3 | 0.3 | 0 | 0 | Agriculture |
| 26 | 0 | 0.3 | 0.3 | 0 | 0 | Forest |
| 27 | 0 | 0 | 0 | 0 | 0 | Grassland |
| 28 | 0 | 0 | 0 | 0 | 0 | Agriculture |
| 29 | 0 | 0 | 0 | 0 | 0 | Lake |
| 30 | 0 | 0 | 0 | 0 | 0 | Forest |
| Total | | 5.3 | 4.5 | 0.8 | | |

**Table S11.** Outcome of the visual interpretation of 30 randomly selected roadless areas in Hungary, showing the assigned ID, the surface of the largest roadless area after road mapping, the sum of all roadless areas created after road mapping, the reduction in size of roadless areas after road mapping, and the main landcover type.

| ID | No. of newly identified roadless areas after road mapping | Roadless area surface before road mapping in ($km^2$) | Roadless area surface after road mapping (largest patch, $km^2$) | Total surface of mapped roadless areas (sum of all new roadless areas after road mapping) | Reduction of roadless area surface after road mapping ($km^2$) | Reduction of roadless area surface after mapping (%) | Landcover type |
|---|---|---|---|---|---|---|---|
| 1 | -1 | 0.4 | 0 | 0 | 0.4 | 100 | Forest |
| 2 | -1 | 0.5 | 0 | 0 | 0.5 | 100 | Agriculture |
| 3 | 0 | 0.4 | 0 | 0.4 | 0 | 0 | Agriculture |
| 4 | 2 | 12.7 | 0.3 | 0.7 | 12.1 | 94.9 | Agriculture |
| 5 | 0 | 8.2 | 0 | 2.3 | 5.9 | 72.2 | Agriculture |
| 6 | -1 | 2.8 | 0 | 0 | 2.8 | 100 | Agriculture |
| 7 | 0 | 1.1 | 0 | 1.1 | 0 | 0 | Agriculture |
| 8 | -1 | 0.1 | 0 | 0 | 0.1 | 100 | Agriculture |
| 9 | 0 | 2.7 | 0 | 1.1 | 1.6 | 58.6 | grassland |
| 10 | 0 | 2.2 | 0 | 2.2 | 0 | 0 | Agriculture |
| 11 | 2 | 6.1 | 0.2 | 0.2 | 5.9 | 97.3 | Agriculture |
| 12 | 0 | 1.9 | 0 | 1.9 | 0 | 0 | Agriculture |
| 13 | 0 | 2.5 | 0 | 0 | 2.5 | 100 | Agriculture |
| 14 | 0 | 1.8 | 0 | 1.8 | 0 | 0 | Agriculture |
| 15 | 0 | 0.4 | 0 | 0.4 | 0 | 0 | Agriculture |
| 16 | 0 | 0.1 | 0 | 0.1 | 0 | 0 | Agriculture |
| 17 | 0 | 4.5 | 0 | 4.5 | 0 | 0 | Agriculture |
| 18 | -1 | 0.6 | 0 | 0 | 0.6 | 100 | Agriculture |
| 19 | 3 | 53.0 | 11.8 | 14.0 | 39.0 | 73.6 | Agriculture |
| 20 | 2 | 7.6 | 0.5 | 0.5 | 7.0 | 92.8 | unknown |

| | | | | | | | |
|---|---|---|---|---|---|---|---|
| 21 | 0 | 0.5 | 0 | 0.5 | 0 | 0 | unknown |
| 22 | -1 | 1.5 | 0 | 0 | 1.5 | 100 | Agriculture |
| 23 | 0 | 29.2 | 0 | 29.2 | 0 | 0 | Lake |
| 24 | 0 | 4.3 | 0 | 2.8 | 1.4 | 33.5 | unknown |
| 25 | 0 | 4.8 | 0 | 2.4 | 2.4 | 50.1 | Agriculture |
| 26 | 0 | 5.7 | 0 | 5.7 | 0 | 0 | Agriculture |
| 27 | 0 | 44.9 | 0 | 44.9 | 0 | 0 | unknown |
| 28 | 0 | 2.4 | 0 | 0.2 | 2.2 | 90.2 | Forest |
| 29 | 2 | 3.1 | 0 | 0 | 3.1 | 99.4 | Agriculture |
| 30 | -1 | 3.5 | 0 | 0 | 3.5 | 100 | Forest |
| Total | | 209.0 | | 116.8 | 92.2 | | |

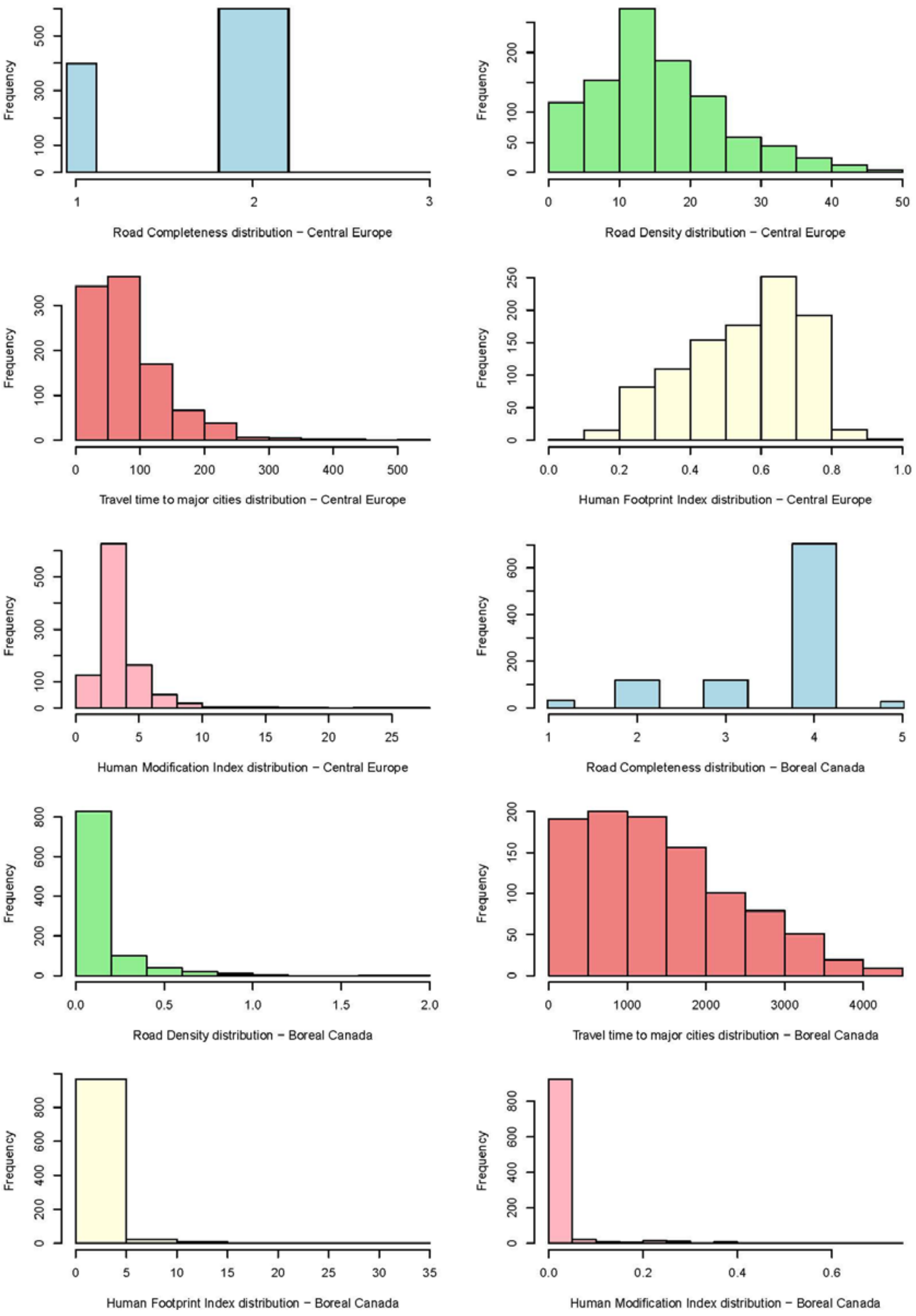


**Figure S1**: Histograms showing the frequency distribution of road completeness, road density, travel time to major cities, Human Footprint Index and Human Modification Index for boreal Canada and temperate Central Europe in the randomly selected 1000 circular plots of 3.14 km$^2$ each (as indicated in Table 1, road completeness refers to plots with all roads completely mapped = 1, plots with roads partially mapped = 2, plots with all roads

unmapped = 3, and plots without road = 4, and plots containing other linear infrastructures = 5), road density (km/km$^2$), travel time to major cities (min), Human Footprint Index (ranging from 0 to 50, low values indicated low human footprint) and Human Modification Index (ranging from 0 to 1, low values indicated low degree of landscape modification by humans).

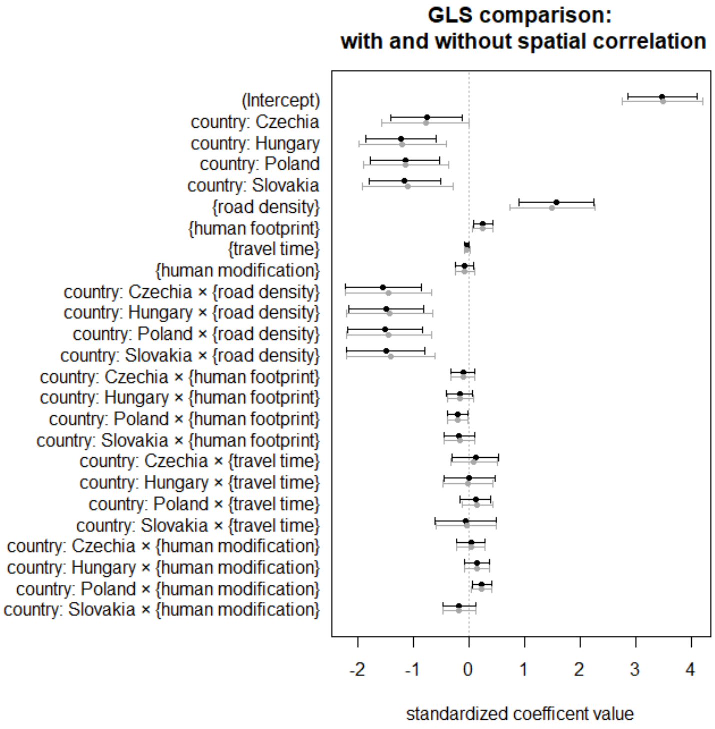


**Figure S2.** Comparison of (standardized) model coefficients of Generalized Least Squares models without (black) and with (grey) accounting for spatial autocorrelation (exponential correlation structure) in the response variable (road completeness). Road completeness encompassed the following three categories, fully mapped = 3, partially mapped = 2, not mapped = 1. The explanatory variables were road density, Human Footprint Index, travel time to major cities and Human Modification Index. The reference country category was boreal Canada.

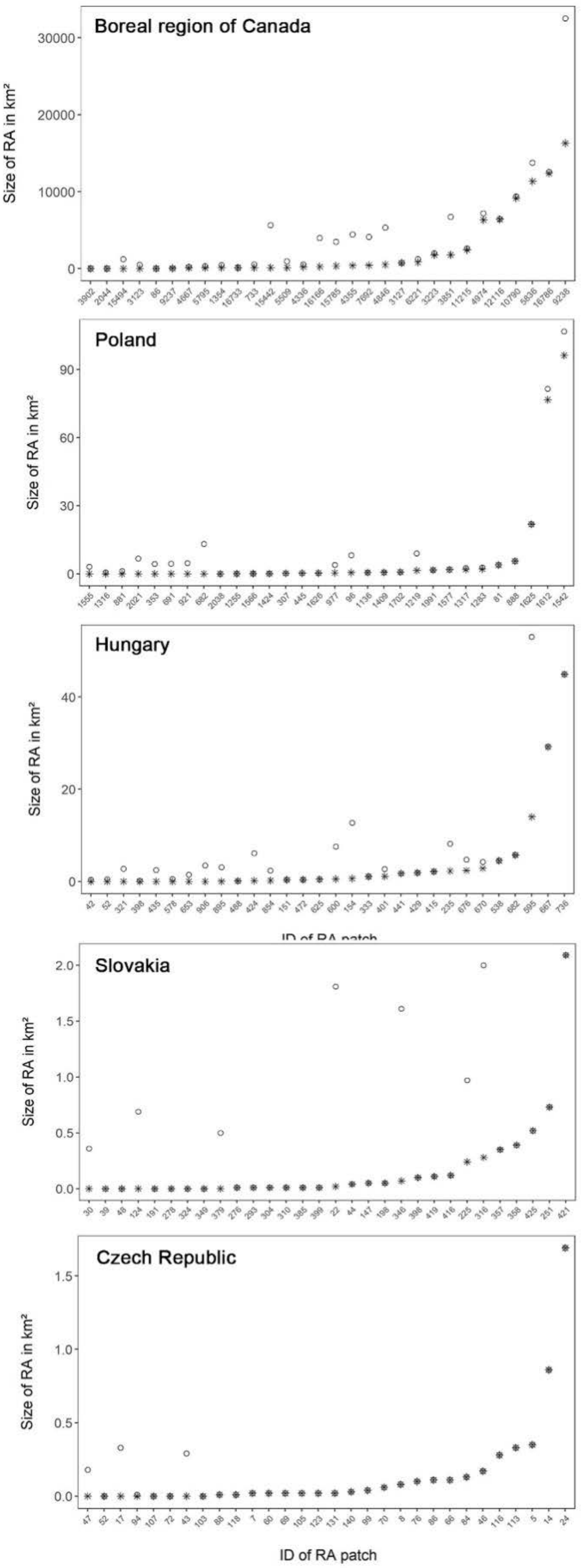


**Figure S3**. Comparison of 30 randomly selected roadless areas in boreal Canada and the four temperate Central European countries before and after visual interpretation and road mapping. The size order was based on the largest roadless area obtained post-mapping. Open circles denote roadless areas before manual mapping, while stars represent the generated largest roadless patches after mapping. Note the different scales.

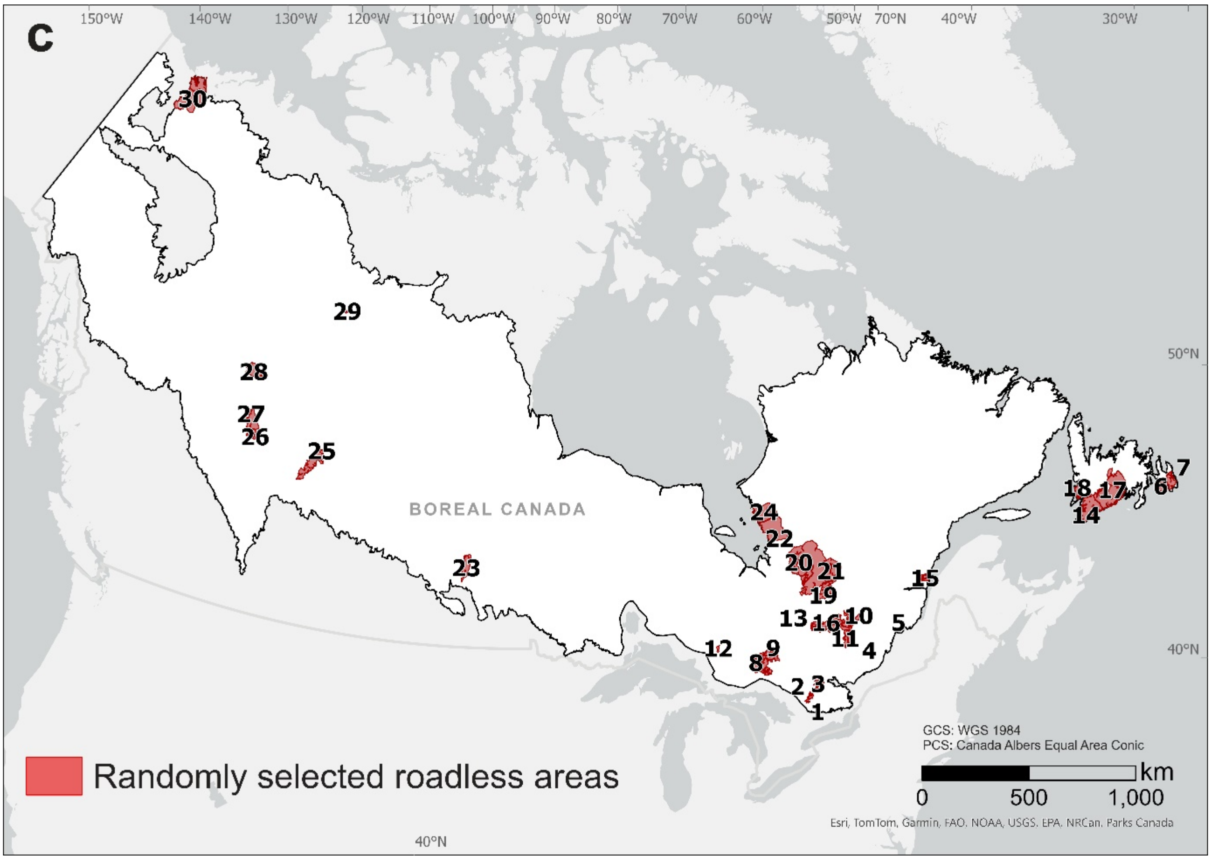


**Figure S4.** The spatial distribution of the 30 randomly selected roadless areas in the boreal region of Canada with their corresponding ID number. See Table S7 for details on each roadless area. This figure was created using ArcGIS Pro 3.2 (https://www.esri.com/en-us/arcgis/products/arcgis-pro/overview).

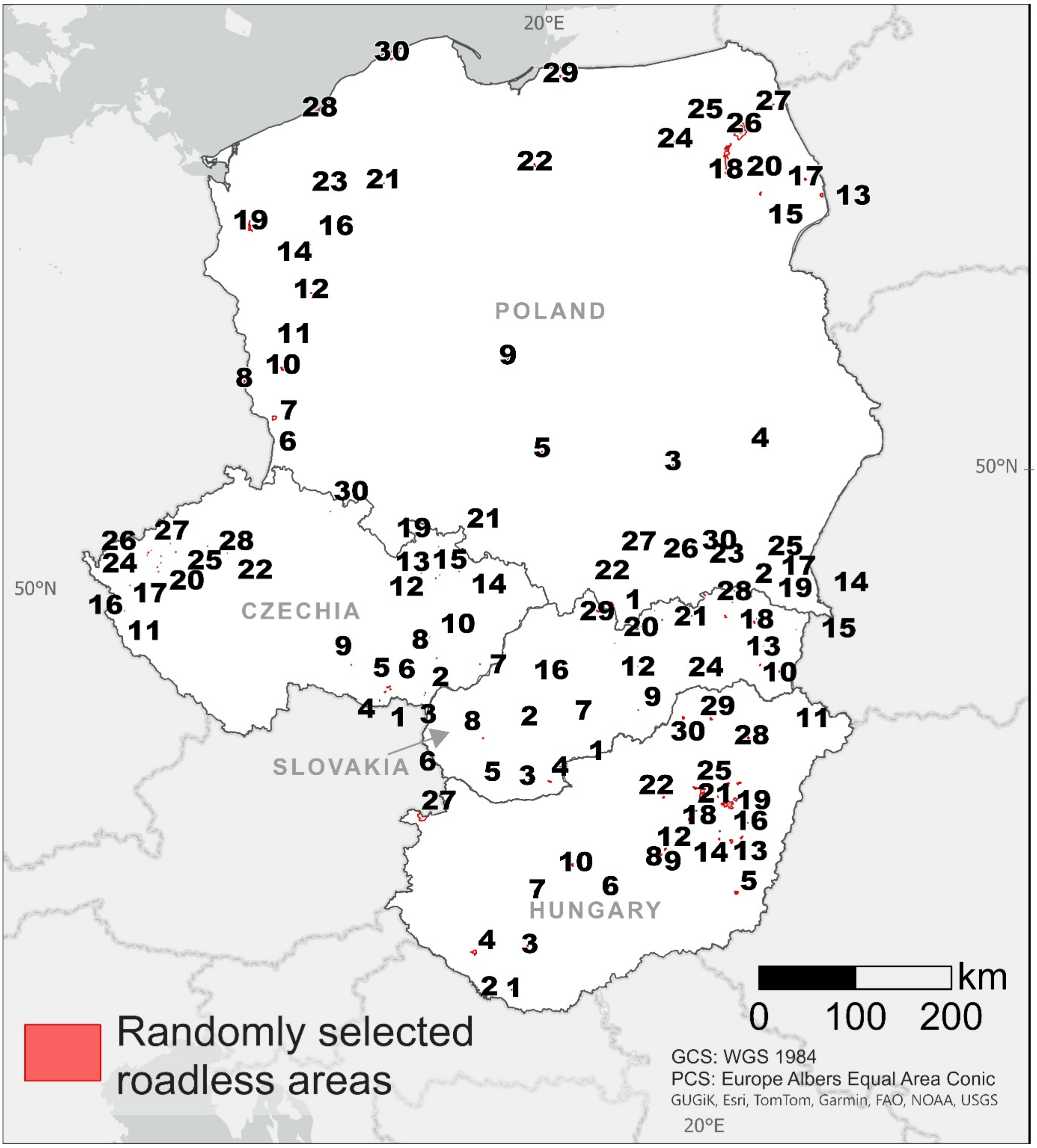


**Figure S5.** The spatial distribution of the 30 randomly selected roadless areas in each of the four selected countries of temperate Central Europe represented by Poland, Slovakia, Czechia, and Hungary are indicated with ID numbers. See Tables S8-S11 for details on each roadless area. This figure was created using ArcGIS Pro 3.2 (https://www.esri.com/en-us/arcgis/products/arcgis-pro/overview).

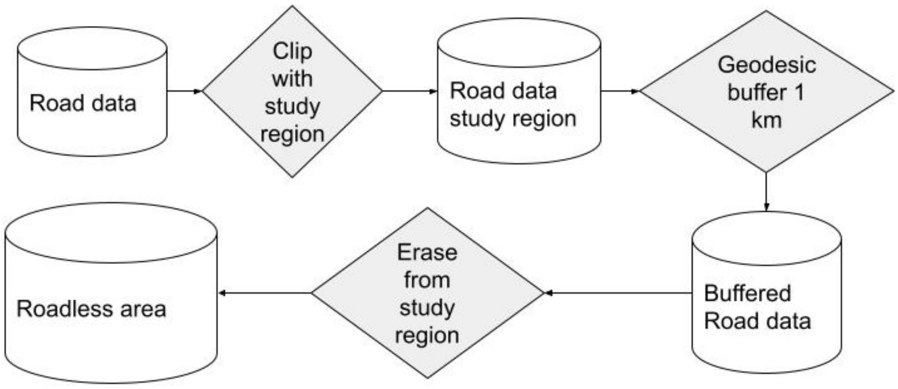


**Figure S6**. Workflow of roadless area calculation.

## References from Supplementary Information


1. OpenStreetMap. Stats. Edits per country. Available at: https://osmstats.neis-one.org/?item=countries [Online: Accessed: 10 April 2022].
2. Geofabrik, http://www.geofabrik.de, OpenStreetMap ODbL. [Online: Accessed: 20 February 2020].
3. OSM Key:highway. https://wiki.openstreetmap.org/wiki/Key:highway" [Online: Accessed: 19 May 2020].
4. Ibisch, P. L. *et al.* A global map of roadless areas and their conservation status. *Science* **354**, 1423-1427 (2016).
5. Natural Earth Data, http://www.naturalearthdata.com/downloads/10m-physical-vectors/10m-lakes/ [Online: Accessed: 15 April 2021].
6. Nelson, Andy. Travel time to cities and ports in the year 2015. figshare. Dataset. DOI: https://doi.org/10.6084/m9.figshare.7638134.v4 (2019) [Online: Accessed: 15 April 2022].
7. Venter, O. *et al.* 2018. Last of the Wild Project, Version 3 (LWP-3): 2009 Human Footprint, 2018 Release. Palisades, New York: NASA Socioeconomic Data and Applications Center (SEDAC). https://doi.org/10.7927/H46T0JQ4. [Online: Accessed: 15 April 2022].
8. Kennedy, C. M., Oakleaf, J. R., Theobald, D. M., Baruch-Mordo, S. & Kiesecker, J. Global Human Modification. figshare. Dataset. https://doi.org/10.6084/m9.figshare.7283087.v1 (2018) [Online: Accessed: 15 April 2020].

9. Hermosilla, T., Wulder, M.A., White, J.C., Coops, N.C. & Hobart, G.W., Disturbance-informed annual land cover classification maps of Canada's forested ecosystems for a 29-year landsat time series. *Canadian Journal of Remote Sensing*, **44**(1), 67-87 (2018).
10. Landcover Europe © European Union, Copernicus Land Monitoring Service <2018>, European Environment Agency (EEA) Available at: https://land.copernicus.eu/pan-european/high-resolution-layers/forests (2022) [Online: Accessed: 15 April 2020].
11. Center for International Earth Science Information Network (CIESIN). Global roads open access data set, Version1 (gROADS), v1 (1980-2010). NASA Socioeconomic Data and Applications Center (SEDAC). Available at: https://sedac.ciesin.columbia.edu/data/set/groads-global-roads-open-access-v1 [Online: Accessed: 15 April 2020].
12. Meijer, J. R., Huijbregts, M. A., Schotten, K. C. & Schipper, A. M. Global patterns of current and future road infrastructure. *Environmental Research Letters* **13**, 064006. Data is available at www.globio.info/download-grip-dataset (2018). [Online: Accessed: 15 April 2020].
13. Canadian Open Data. National Road Network (NRN). Available at: https://canadiangis.com/national-road-network-nrn-canadian-open-data.php [Online: Accessed: 17 April 2020].
14. EuroGeographics. Open Maps for Europe. Available at: https://eurogeographics.org/open-maps-for-europe/open-maps-for-europe-progress/ (2022) [Online: Accessed: 20 April 2022].
